# How the ecological structure affects the aesthetic atmosphere of the landscape: Evaluation of the landscape Beauty of Xingqing Palace Park in Xi’an

**DOI:** 10.1371/journal.pone.0302855

**Published:** 2024-05-15

**Authors:** Weiwei Jin, Wenyu Miao

**Affiliations:** Philosophy Dept, Sch Humanities Social Sci, Xi’an Jiaotong University, Xi’an, Shaanxi, Peoples R China; Shahid Beheshti University, IRAN, ISLAMIC REPUBLIC OF

## Abstract

In landscape appreciation, what tourists directly perceive is the atmosphere of the landscape. This paper introduces the concept of “Ecological Structure” from Gernot Böhme’s theory of atmospheric aesthetics into the assessment of landscapes, utilizing atmosphere as a bridge between horticultural ecology, aesthetics, and culture. It examines the relationship between the objective environment and subjective perception. This study conducted a field survey of Xingqing Palace Park and selected the waterside plant landscape that directly reflects the atmosphere of the royal garden as the research object. In the first stage of this study, Scenic Beauty Estimation was used to evaluate the overall beauty of 32 landscape units; in the second stage, the Delphi method and Analytic Hierarchy Process were used to evaluate the ecological structures that affect the garden landscape atmosphere; in the third stage, the two evaluation results of the Kendall’s W concord coefficient test Analytic Hierarchy Process and Scenic Beauty Estimation have high consistency, which shows that the atmosphere is great value to the beauty of the landscape. This study provides designers with a means to create a garden atmosphere using ecological structures and provides new ideas for landscape design.

## 1 Introduction

Sensibility is primarily concerned not with the objects people perceive, but with the atmosphere. The field of atmospheric research has mainly focused on the theory of environmental aesthetics, but in recent years, practical applications in landscape design have steadily increased. Many environmental aesthetes, most prominently Böhme, believe that atmosphere is the unity of objective factors with subjective physical sensations and mental perceptions [1, 2]. It refers to material forms and non -material environments such as the senses, emotional dimensions, temporality and memory [3]. When people appreciate a certain natural scenery, they do not simply appreciate the objective nature or the subjective emotional projection, but a mediators of communication between the subject and the object, that is atmosphere [4]. Sumartojo talks about the impact and concept of atmosphere as more than just the sense of places and events [5], it is about adjusting space [6], folding space and time [7]. In terms of the impact of the atmosphere, according to Adams and Brown et al, the attmosphere is valuable in improving the quality of places and landscapes [8, 9]. Others have proposed similar ideas: combining atmosphere with design, art configuration, scenography, immersive performance, and visual arts to provide a key to theoretical and aesthetic guidance for landscape design professionals and citizens alike [6, 10]. However in the current study, few assessment tools have been identified and validated to evaluate the atmospheric quality of landscapes. Phil Jones and others introduced the urban architectural landscape and it’s atmosphere through case study [11], and Minna explored the aesthetic atmosphere of urban squares and their inspiration through questionnaire [10]. These papers only evaluate the overall atmosphere of the landscape, and fail to analyze the components that affect the atmosphere. In addition, the phenomenon of aesthetics atmosphere sometimes cannot accurately and appropriately express the local culture [12], as well as the subtle power of atmosphere has a positive or negative impact on emotions and cognition of people [13]. More importantly in the process of creating atomospere, the current landscape design focuses on the picturesque, affective experience, and overall presentation of atmosphere in the construction of atmosphere, thus ignoring elements such as ecological health and cultural expression within the site. Thus, landscape design professionals need to find a design concept that balances ecology and aesthetics to create an atmosphere with an attractive and positive place.

In Böhme’s aesthetics of atmosphere, the concept of ecological structure is repeatedly emphasized. However, in the theory and application of environmental aesthetics, this concept has not been thoroughly explored [14, 15]. “Ecological structure” is different from “ecosystem”, in that it adds humanistic factors, such as culture, economy, law, etc., which means that they seek a kind of “social and natural sciences” [16, 17]. However, how to conceptually understand “ecological structure”: is the future protection of natural and cultural landscapes based on “social and natural sciences”? The term “ecological structure” was introduced by Böhme and Schramm in their *Soziale Naturwissenschaft*, which include both summary of the living world and abiotic factor limited to social conditions [18]. The Structure is an ecological unit of the ecological representation of a complete ecosystem with ecological appearance, such as a island, grassland, etc. “Ecological Structures” are “not determined by natural boundaries like ecosystems alone; rather, they are delineated by social and political boundaries, specifically property boundaries [15]”. For example, the urban parks are a good illustration of “Ecological Structure”: the natural boundaries of the park are composed of objects, and in terms of space, it is defined by the contours of streets and houses. Thus, an ecological structure is not only formed through natural sequences, but must also be created by social elements-through culture, economy, and labor. In addition, it natural landscapes or objects belong to “social and natural sciences”, that is “ecological structure”, so they mainly appear as various atmospheres [19]. The ecological construction of the view of nature, its focuses on human feelings about their situation, in other words, it is focuses on “how we feel and perceive, that is, how we perceive nature”. In short, ecological structure is the elements that constitute of atmosphere, which is helpful to provide a practical quantitative path for landscape design, people perceive ecological structure through sensory experiences and use to create attractive landscape.

Landscape design still favored picturesque scenery, which was also influenced by citizens, as many considered ecological landscape too cluttered and wild [20, 21]. Many environmental aestheticians share a similar view, arguing that picturesque landscape “should be rejected and replaced by a practice of valuing nature based on a clear, scientific understanding of the environment [22]”. Picturesque landscapes mainly engage the visual senses, other senses are generally not involved [23–25]. For example, the public, including designers, artists, philosophers, and citizens, generally enjoy color, shape, and form in scenery. There was discussions of the neat grassland, spherical shrub, porportion, symmetry, etc. as if the landscape was like a painting [26]. Therefore, landscape designers usually use the visual methods of landscape picture [27] to develop design ideas. However, this approach can sometimes neglect the balance of ecosystem and the involvement of sensory aesthetic. This paper argues, in line with the aesthetic theories of Gernot Böhme and others, landscape designs needs to change the aesthetic thinking of designers and citizens to address the gap of visual beauty and sensory engagement in landscape design through the use of “ecological structures”. Furthermore, we attempt to quantify the relationship between “ecological structure” and “atmosphere” through quantitative methods.

As an “ecological structure”, garden landscape is a typical representative of atmosphere creation [16]. On the one hand, gardens are composed of objective and realistic natural objects including streams, flowers, meadows, forests, and so on [28]. On the other hand, human factors include historical, cultural, economic and other factors, intangible elements such as, natural sounds, light, and shadow are all used as means to create an atmosphere, and the atmosphere moves toward ecological aesthetics. Different ecological structures in the garden landscape generate different atmospheres, such as gardens with a cosy atmosphere, waters with a mysterious atmosphere, and bamboo forests with a meditation atmosphere. The promotion of atmosphere in the garden is conducive to the return of nature to physical, emotional and imaginative participation, and at the same time pays attention to the relationship between the aesthetics of nature and ecology and culture [16, 29]. The desirable atmosphere of the garden is that people’s body and mind are integrated into the “nature”, and then feel the ecological and cultural beauty of the garden itself. In Chinese classical gardens, nature and human feelings are integrated, and subjective perceptions are mirrored in the natural landscape [30, 31]. The appearance of plants under the influence of ecological quality gives people an intuitive perception, so that people step into the virtual and real combination in the garden artistic conception (the reality of the landscape and the atmosphere of the virtual). As a royal garden during the prosperous Tang Dynasty, Xingqing Palace Park is based on the ruins of the Tang Dynasty. Xingqing Palace Park is built along the lake, and its main landscapes are waterfront plant landscapes. The waterfront plant landscape in the park uses a variety of design techniques, such as master view, supporting view, barrier view, opposite view, etc., to cleverly organize the waterfront space and create different atmospheres of different themed gardens [32, 33].

Therefore, starting from the atmosphere, it is very important to analyze how the “ecological structure” of the landscape affects the tourists’ aesthetic evaluation of the plant landscape. Firstly, atmosphere is the link between objective factors and subjective series of bodily sensations. Gernot Böhme proposed that this means that atmosphere is a factor between the subject and the object, an intermediary between the both sides [16]. Therefore, the research about the atmosphere aesthetics of the garden, on the one hand, it can be from the perspective of visitors’ perception aesthetics; on the other hand, it can be from the perspective of the work aesthetics of the landscape. Secondly, at present, the main methods in the field of plant landscape evaluation are analytic hierarchy process [34–36], scenic beauty estimation method [37–39], fuzzy logic system [40–42], and principal component analysis [43–45] and so on. This paper selects the scenic beauty estimation method and analytic hierarchy process according to the perspective of atmosphere aesthetics research. SBE is to quantify the psychological quantitative value of the evaluator’s feelings or perceptions from the perspective of tourist’s subjective perceptions [46]. The advantage of this method is that it quantifies the tourists’ subjective aesthetic evaluation of the landscape and thus reflects the aesthetic value of the landscape, and the evaluation results are universal and practical. On the contrary, AHP is a systematic hierarchical analysis method combining qualitative and quantitative from the perspective of the objective [47], from the aesthetic perspective of the objective reality of the landscape works, which can mathematize the thinking process of decision-making with less quantitative information [48]. As a result, it can refine the ecological structure of the landscape, providing a simple approach to the complex decision-making problem of creating atmosphere with multiple medium factors or unstructured features.

The value of garden atmosphere is widely recognized in the theory of environmental aesthetics. In this paper from the perspective of ecological construction, based on the overall grasp of the aesthetic atmosphere of Xingqing palace Park in Xi’an City, we take the waterfront plant landscape as the research object, using SBE and AHP to evaluate and analyze the influence of the ecological construction on the creation of atmosphere. The main purposes of this article are threefold: first, to conduct a quantitative analysis of the ecological structure, which is a component of landscape atmosphere, from the perspective of atmospheric aesthetics, so as to increase understanding of the constituent elements of landscape aesthetic atmosphere and promote the development of atmospheric theory in the field of environmental aesthetics. Second, to establish a research path for the impact of ecological structure on landscape aesthetic atmosphere through SBE, AHP, and principal component analysis, and explore the degree of impact of ecological structure on aesthetic atmosphere. Third, to address the problem of unmet emotional needs in modern landscape design, and provide references for landscape planners and garden designers in utilizing the theory of “ecological structure” to create attractive atmospheric landscapes.

## 2 Materials

### 2.1 Research area

The main reason for choosing Xingqing Palace Park as the research object is that China had strong national power and economic prosperity during the Tang Dynasty. Xingqing Palace, as a royal garden and political center built at this time, demonstrated the atmosphere of the Golden Dynasty. During the historical changes, Xingqing Palace was built and renovated many times. Although it cannot compare with the prosperity of the Tang Dynasty, it has always been a holy place to visit. From the 1950s to the 1980s, Xingqing Palace Park was built on the site of Xingqing Palace in the Tang Dynasty and was successively restored. 2020–2021 Xi’an Municipal Government of Xingqing Palace Park and once again optimized the renovation of Xingqing Palace Park, which was basically able to revive its magnificent and majestic atmosphere. Xingqing Palace Park is currently located in the north side of Xianning West Road outside the East Gate of Xi’an City, in the original Xingqingfang of Chang’an City (Xi’an was formerly called Chang’an) [49]. The park is designed with freehand brushwork, taking the trend of high in the northwest and low in the southeast. The three mountains are planted with trees, and there are three islands in the lake. With Longchi as the center, the Agarwood Pavilion and Nanxun Pavilion are arranged according to the original Xingqing Palace. This survey conducted field survey of Xingqing Palace Park and based on consulting professionals, selected waterfront landscapes such as, ChangQing Pavilion, Yuanyang Bridge, Wulong Lake, Nanxun Pavilion, and Longchi lotus landscape as the main research objects, and representative and comprehensive plant landscapes were selected as research materials.

### 2.2 Data collection

In order to enhance the beauty of the pictures of the scenic spots and increase the richness of the colors, on the basis of consulting professionals, the sample photos were collected in the summer with the largest flow of people in Xingqing Palace Park, and were taken in sunny weather from 9:00 to 16:00 on May 15, 2023 to August 15, 2023.

Using the Braun-Blanquet sample survey method [50], 32 typical and representative waterfront plant landscape units were selected and randomly numbered, and were photographed from close-up, medium-range, and long-range based on Chinese gardening techniques. To improve the level of picture analysis, shoot from multiple angles and directions at the same time, and record plant species, plant quantity and other relevant parameters in the landscape unit in real time. The sample plot standard is 25m×25m. If roads and watersides are encountered, roads and water systems will be used as natural boundaries.

A total of 296 photos were taken in this study, from which 64 photos were selected that better reflected the 32 shooting points of the waterfront plant landscape, and 2 photos were taken from each landscape shooting location.

## 3 Research methods

### 3.1 Scenic Beauty Estimation(SBE)

Based on the 64 photographs selected from the survey sample site, 50 teachers and students from the College of Humanities and Social Sciences of Xi’an Jiaotong University were invited to rate the photographs through questionnaire from Landscape Design major in the Department of Art and Environmental Aesthetics major in the Department of Philosophy, and 50 on-spot tourists, including 50% females. The scoring criteria were based on a 5-point scale, ranging from 1 to 5. A score of 1 represents a poor plant landscape effect, 2 represents an average plant landscape effect, 3 represents a not-so-good plant landscape effect, 4 represents a good plant landscape effect, and 5 represents a plant landscape with high aesthetic beauty. Subsequently, the scores of each group of 32 landscape units were summarized using Excel software, and the scores were calculated using the scientific and reliable method of Scenic Beauty Estimation. The degree of scenic beauty calculation formula was:

$$\begin{array}{c} S_{i}=5\times \sum_{j=1}^{5}(\frac{n_{ij}}{N}\times j) \end{array}$$ (1)

In this formula, the scenic beauty of plant landscape *i*; N is the total number of people who evaluated(49); and *n*_*ij*_ is the number of people who scored *j* for plant landscape *i*. Therefore, this evaluation used the SBE to calculate the evaluation results.

### 3.2 Analytic Hierarchy Process (AHP)

#### 3.2.1 Constructing evaluation system

According to previous literature, atmosphere is the first topic that tourists feel, and it is the key element that triggers emotion and establishes human-scape connection [3, 51]. Ecological structure is the foundation of garden atmosphere, including ecological natural elements such as plants, vegetation, water bodies, etc., and human elements such as artistic configuration, cultural history, etc., even the subtle leaf forms can affect the atmosphere [15, 52]. Therefore, this paper adopts a two-step method with Delphi and AHP method to understand which factors in the ecological structure have the most influence on the landscape atmosphere. Participating experts include 30 experts in landscape design, environmental aesthetics, and atmosphere aesthetics, including senior engineers in landscape design of design institutes, senior engineers in water conservancy engineering, professors and associate professors in aesthetics, and have rich experience in gardens appreciation. Based on the collection of experts’ questionnaires and the application of the factor analysis method, we finally formulated an evaluation index system for the ecological structure of the waterfront plant landscape in Xi’an Xingqing Palace Park. This system encompasses target, criterion, and index layers (Table 1).

**Table 1:** Evaluation index system of waterfront plant landscape ecological structure in Xingqing Palace Park, Xi’an.

Target layer	Criterion layer	Index layer	Index meaning
Waterfront Plant Landscape of Xingqing Palace Park in Xi’an(A)	Ecology element(B1)	Species diversity (C1)	Number of species in waterfront planted landscapes
		Community stability (C2)	Ecological balance of communities in waterfront planted landscapes
		Underground vegetation coverage (C3)	Percentage of vertical projection area of branches and canopy of grass and irrigation plants in sample plot area
		Locality of plant communities (C4)	Regional characteristics of plant appearance in waterfront planted landscapes and adaptation to the climatic environment of the site
		Ecological health of water bodies (C5)	Observed water clarity and reflection clarity
	Aesthetics element(B2)	Plant color diversity (C6)	Number of plant colors in the park’s plant landscape
		Plant shape and texture (C7)	Overall plant form and texture in park plant landscapes
		Plant visual hierarchy (C8)	Number of levels of vertical differentiation of park plant landscapes observed by the human eye
		Plant neatness (C9)	Consistent expression of plant color, species, level, texture and canopy line
		Harmony between plants and water bodies(C10)	Degree of harmony between plants and the water environment
		Plant peculiarities (C11)	The degree of oddity in people’s visual perception in vision
		Seasonal plant ornamental period (C12)	The length of the ornamental cycle of seasonal plants in planted landscapes
		Diversity of botanical art configurations (C13)	Botanical artistry paired with sculpture, architecture, drawings, and other types of quantities
		Plant aroma perception (C14)	Degree of perception that plants have an aroma
	Culture element(B3)	Positive plant culture meanings (C15)	Plants have positive connotations in Chinese culture
		Plant cultural characteristics (C16)	Adaptation of Plant Cultural Characteristics traits to park types
		Plant growth years (C17)	Year of growth of major plants
		Proportion of endangered and valuable plants (C18)	Proportion of the number of endangered and valuable plants

Taking the elements of landscape aesthetics as an example, the multicollinearity among the indicators in the evaluation system was tested. This study uses confirmatory factor analysis method to simplify the structure of the landscape aesthetic elements scale. Principal component analysis is used as the method to extracting factors, eigenvalue greater than 1 are used as the criterion for selecting factors. The orthogonal rotation method used as a, specifically the varimax rotation method, was adopted as the rotation technique. The KMO measurement found that the KMO value of the scale was 0.617, indicating that there are potential shared factors and factor analysis can be performed. Factor analysis extracted a total of three factors, with the cumulative variance contribution rate of 62.44% (see Table 2). Based on the results of the factor analysis, the three factors have been labeled as “landscape perception,” “landscape intuition,” and “landscape sensation.” Among them, landscape perception includes appreciation of plant color diversity, plant shape and texture, plant visual hierarchy, plant uniqueness, seasonal plant viewing period, and diversity of botanical art configuration. Landscape intuition includes appreciation of plant uniformity and harmony between plants and water. Finally, Landscape sensation includes appreciation of plant aroma perception. Ecological structure integrates perceptual, intuitive, and sensory psychological factors to influence people’s judgment of the aesthetic atmosphere of landscapes.

**Table 2:** Factor loading matrix of landscape aesthetic elements.

	Landscape perception	Landscape intuition	Landscape sensation	Communality
Plant color diversity	0.67	0.23	-0.49	0.26
Plant shape and texture	0.67	-0.08	-0.47	0.32
Plant visual hierarchy	0.70	0.15	-0.13	0.46
Plant uniformity	0.42	0.62	0.13	0.41
Harmony between plants and water bodies	0.32	0.68	0.40	0.28
Plant peculiarities	0.41	-0.15	-0.04	0.81
Seasonal plant viewing period	0.53	-0.67	0.04	0.27
Diversity of botanical art configuration	0.63	-0.31	0.30	0.42
Plant aroma perception	0.46	-0.18	0.78	0.14
Rotated Eigenvalues	2.72	1.54	1.36	
Variance Contribution Rate(%)	30.21	17.08	15.15	
Cumulative Variance Contribution Rate(%)	30.21	47.29	62.44	

After introducing factors into the regression model, the analysis results of multiple linear regression are as follows:

Based on the regression analysis results presented in Table 3, we can see that factors f1 and f2 have a significant positive impact on landscape aesthetic elements, which means that the results of this factor analysis can be used to effectively explain the landscape aesthetic elements.

**Table 3:** Multivariate linear regression model of landscape aesthetic elements based on factor analysis of landscape aesthetic elements.

	Coefficient	Variance
Landscape perception	0.2792412**	0.098851
Landscape intuition	0.1815823+	0.0988146
Landscape sensation	0.0125104	0.0919673
Intercept	4.333333***	0.0903023

Note: (1) +p<0.1, *p<0.05, **p<0.01, ***p<0.001. (2) Each coefficients in the table is an unstandardized coefficients.

#### 3.2.2 Constructing two-by-two comparison judgement matrix

Based on the construction of the comprehensive evaluation model, this study consults 30 experts in the direction of landscape design and environmental aesthetics through the two-round Delphi method to assign importance scores to the indicators in the evaluation system of the waterfront plant landscape of Xi’an Xingqing Palace Park. The mean difference of importance scoring is created using Saaty’s 1–9 scale method (Table 4) to create a judgment matrix model [53], where the scale is equal as follows: assuming that *Z*_*ij*_ and *Z*_*jk*_ for the same level in the importance of the two indicators of the mean, if *Z*_*ij*_ − *Z*_*jk*_ = 0, then *Z*_*ij*_ and *Z*_*jk*_ are equally important and Saaty scale is equal to 1; If 0.25 < *Z*_*ij*_*Z*_*jk*_ < 0.50, then *Z*_*ij*_ is slightly important than *Z*_*jk*_ and Saaty scale is taken as 3; If 0.75 < *Z*_*ij*_*Z*_*jk*_ < 1.00, then Zij is pretty important than *Z*_*jk*_ and Saaty scale is taken as 5; If 1.25 < *Z*_*ij*_ − *Z*_*jk*_ < 1.50, then *Z*_*ij*_ is significantly important than *Z*_*jk*_ and Saaty scale is equal to 7; If Zij − *Z*_*jk*_ > 1.75, then *Z*_*ij*_ is absolutely important than *Z*_*jk*_ and Saaty scale is equal to 9; If the mean difference of the importance assignment is between two levels, the Saaty scale is 2, 4, 6, 8 accordingly. Also, the study used Yaahpv12.10 software to calculate the weight values and consistency ratio CR for each level of single sorting and perform a consistency test. If the CR value of the judgment matrix in the calculation result is <0.1, it’s mean that the consistency test passed; otherwise, the consistency test fails. It is necessary to return to the calculation step of the index weights to recalculate until the consistency test passes.

**Table 4:** The 1–9 scale and the index scale in the AHP.

1–9 Scale	Definition
1	Equal importance
3	Moderate importance
5	Strong importance
7	Very Strong or demonstrated importance
9	Extreme importance
2,4,6,8	Used as a compromise between the above criteria
Reciprocal of above	If activity i has one of the above nonzero numbers assigned to it when compared with activity j, then j has the reciprocal value when compared with i.

#### 3.2.3 Score and ranking criteria

The evaluation factors consist of 2 kinds of factors: Quantitative and Qualitative. Quantitative indicators include species diversity, underground vegetation coverage, plant growth years, locality of plant communities, plant color diversity, plant visual hierarchy, seasonal plant ornamental period, diversity of botanical art configurations, and proportion of endangered and valuable plants. Species diversity, underground vegetation coverage, plant growth years, locality of plant communities, and proportion of endangered and rare plants are statistically calculated based in to the plant species, number of plants, area, and frequency of occurrence applied in the design space unit. The indicators of plant color diversity, plant visual level, and diversity of botanical art configurations are counted according to the number and type of colors, art configurations, and levels appearing in the waterfront plant landscape. Seasonal plant viewing period index is calculated based on the plant viewing characteristics and best viewing time for 4 viewing types: flower viewing, leaf viewing, fruit viewing, or other viewing types. Based on the quantitative indicator scoring criteria (Table 5), the study directly scores the 32 landscape sample photos based on the research statistics and references.

**Table 5:** Evaluation index system of waterfront plant landscape ecological structure in Xingqing Palace Park, Xi’an.

Quantitative Indicator	Scoring Criteria				
	5	4	3	2	1
Species Diversity	Plant Species ≥ 7	5–6 Plant Species	3–4 Plant Species	2 Plant Species	1 Plant Species
Underground Vegetation Coverage	Underground Vegetation Coverage>80%	Underground Vegetation Coverage 60–80%	Underground Vegetation Coverage 40–60%	Underground Vegetation Coverage20–40%	Underground Vegetation Coverage <20%
Plant Growth Years	Years Of Major Plant Growth ≥ 5 Years	Main Plant Growth Years 4–5 Years	Main Plant Growth Years 3–4 Years	Main Plant Growth Years 2–3 Years	Year Of Major Plant Growth <2 Years
Locality Of Plant Communities	Proportion Of Native Plants >80%	60–80% Of Native Plants	40–60% Of Native Plants	20–40% Of Native Plants	60–80% Of Native Plants
Plant Color Diversity	Main Plant Colors ≥ 5	4 Main Plant Colors	3 Main Plant Colors	2 Main Plant Colors	Main Plant Colors ≤ 1
Plant Visual Hierarchy	Plant Visual Hierarchy ≥ 5 Layers	4 Layers Of Plant Visual Hierarchy	3 Layers Of Plant Visual Hierarchy	2 Layers Of Plant Visual Hierarchy	Plant Visual Hierarchy ≤ 1 Layer
Seasonal Plant Ornamental Period	Seasonal Plant Ornamental Period >80 Days	Seasonal Plant Ornamental Period Of 60 80 Days	Seasonal Plant Ornamental Period Of 40 60 Days	Seasonal Plant Ornamental Period 20 40 Days	Seasonal Plants Ornamental Period <20 Days
Diversity Of Botanical Art Configurations	Number Of Art Configurations ≥5	Number Of Art Configurations 4	Number Of Art Configurations 3	Number Of Art Configurations 2	Number Of Art Configurations ≤1
Proportion Of Endangered And Valuable Plants	Proportion Of Endangered And Valuable Plants >40%	Proportion Of Endangered And Valuable Plants 30–40%	Proportion Of Endangered And Valuable Plants 20–30%	Proportion Of Endangered And Valuable Plants 10–20%	Proportion Of Endangered And Valuable Plants <10%

Qualitative indicators include community stability, water ecological health degree, plant shape and texture, plant neatness, plant peculiarities, harmony between plants and water bodies, plant aroma perception, positive plant culture meanings, plant cultural characteristics. Qualitative indicators are quantified through the expert scoring method. The score of each indicator is set as 5, 4, 3, 2, and 1. The higher the score, the better the evaluation factor corresponding to the plant landscape. In this study, the qualitative factors of each plant landscape picture were determined by 30 experts in landscape design and environmental aesthetics, and then averaged.

The comprehensive score was calculated through the landscape comprehensive evaluation index method, i.e., *B* = ∑ F_*i*_ × X_*i*_. B is the comprehensive evaluation index of ecological structure of the waterfront plant landscape in Xi’an Xingqing Palace Park; *X*_*i*_ is the weight value of *i* evaluation factor; F_*i*_ is the score value of the waterfront plant landscape in Xi’an Xingqing Palace Park, under *i* evaluation factor. Finally, use the formula *CEI* = S/S_0_ × 100% to determine the grade of the waterfront planted landscape. Among them, *CEI* is the comprehensive score of plant landscape quality; *S* is the actual score; *S*_0_ is the ideal plant landscape quality value, which is obtained by taking the highest level of each factor and superimposing it by multiplying the weights. Based on the CEI calculation results, the quality of Xi’an Xingqing Palace Park’s waterfront plant landscape ecological structure was divided into five levels through the difference percentage grading method, which are High quality (100% ∼ 80%), Preferably (79% ∼ 60%), Moderate (59% to 40%), Poor (39% to 20%), and Inferior (19% to 0%) (Table 6).

**Table 6:** Park waterfront plant landscape quality evaluation and grading standards.

Plant landscape quality grade	Description of plant landscape quality characteristics
Inferior	Very simple landscape ecology, very low landscape aesthetics, very simple landscape culture
Poor	Simple landscape ecology, low landscape aesthetics, simple landscape culture
Moderate	Complex landscape ecology, high landscape aesthetics, high landscape culture
Preferably	More complex landscape ecology, higher landscape aesthetics, more complex landscape culture
High quality	Very complex landscape ecology, very high landscape aesthetics, very complex landscape culture

### 3.3 AHP and SBE in combination

#### 3.3.1 The Kendall coefficient of concordance test

The Kendall’s Coefficient of Concordance was used to test whether the ranking results of the two evaluation methods AHP and SBE are consistent. Import the ranking information of the evaluation results of the two methods into SPSS24.0, first select Nonparametric Test, then select the K Related Samples module, next select the Kendall’s coefficient of concordance test function, and finally complete the test.

#### 3.3.2 Standardized processing

After Kendall’ s W concord coefficient test, if there is consistency between the rank evaluation results of the two methods SBE and AHP, then the standard deviation method needs to be used to standardize the results. The score standardization formula is

$$\begin{array}{c} Z_{ij}=\frac{X_{ij}-\bar{X_{i}}}{S_{i}} \end{array}$$ (2)

*Z*_*ij*_ is the standard score of *j* plant landscape in the *i* evaluation method; *X*_*ij*_ is that score of the *j* plant landscape unit in the *i* evaluation method; ${\bar{X}}_{i}$ is the average score of all the plant landscapes in the *i* evaluation method; S_*i*_ is the standard deviation of all plant community scores in the *i* evaluation method.

#### 3.3.3 Summary score

The results were aggregated from the standardized scores of the AHP and SBE methods assessment results. The formula for the composite score is:

$$\begin{array}{c} T_{j}=\sum_{i=1}^{k}X_{ij} \end{array}$$ (3)

In the formula: T_*j*_ represents the total score of *j* plant landscape unit sample under *k* evaluation methods.

## 4 Results and analyses

### 4.1 Analysis of AHP evaluation results

This paper uses Yaahpv12.10 software to get the weight values of the indicators at all levels of the ecological construction of waterfront plant landscape in Xingqing palace Park, Xi’an (Table 7). As can be seen from Table 7, in the evaluation system of ecological structure of waterfront plant landscape in Xingqing palace Park, Xi’an, the weight size of the criterion layer is ecological element (0.59) > aesthetics element (0.25) > cultural element (0.16). The highest ranking indicator layers are regionality of plant communities and ecological health of water bodies with weights of 0.22 and 0.15 respectively. The harmony between plants and water bodies and the perception of plant aroma are the most important index layer elements of aesthetic elements, with their weights being 0.07 and 0.05 respectively. Among the cultural element, two index-level elements perform very prominently. One is the positive plant culture meanings with a weight of 0.09, and the other is the plant cultural characteristics with a weight of 0.05. These two index-level elements even exceed many sub-indicators of ecological element and aesthetic element. Based on the weights of the indicators and the actual measured values of the indicators in Xingqing palace Park, we calculated and collated the results of the evaluation of ecological structure of the park’s plant landscape in Xingqing palace Park (Table 8). Analyzing the data presented in Table 8, it can be found that among the 32 plant landscape units studied, there are 5 high-quality landscapes, 18 preferably landscapes, 9 moderate landscapes, and no poor and low-quality landscapes. This indicates that overall, the waterfront plants landscape of Xingqing Palace Park is relatively good.

**Table 7:** Evaluation weight values of ecological structure of waterfront plant landscape in Xingqing Palace Park, Xi’an.

Target layer	Criterion layer	Criterion layer weight	Index layer	Index layer weight	Combined weights	Combined weight ranking	consistency test
Evaluation index System of Waterfront plant Landscape factors in Xingqing Palace, park, Xi’an City (A)	Ecological element (B1)	0.59	Species diversity (C1)	0.19	0.11	3	CR = 0.03<0.1 Consistency test passed
			Community stability (C2)	0.13	0.08	5	
			Underground vegetation coverage (C3)	0.07	0.04	9	
			Locality of plant communities (C4)	0.37	0.22	1	
			Ecological health of water bodies (C5)	0.25	0.15	2	
	Aesthetics element (B2)	0.25	Plant color diversity (C6)	0.05	0.01	14	CR = 0.04<0.1 Consistency test passed
			Plant shape and texture (C7)	0.10	0.03	12	
			Plant visual hierarchy (C8)	0.07	0.02	13	
			Plant neatness (C9)	0.04	0.01	16	
			Harmony between plants and water (C10)	0.26	0.07	6	
			Plant peculiarities (C11)	0.03	0.01	18	
			Seasonal plant ornamental period (C12)	0.14	0.04	10	
			Diversity of botanical art configurations (C13)	0.12	0.03	11	
			Plant aroma perception (C14)	0.19	0.05	8	
	Cultural element (B3)	0.16	Positive plant culture meanings (C15)	0.56	0.09	4	CR = 0.05 <0.1 Consistency test passed
			Plant cultural characteristics (C16)	0.23	0.05	7	
			Plant growth years (C17)	0.08	0.01	15	
			Proportion of endangered and valuable plants (C18)	0.05	0.01	17	

**Table 8:** AHP Evaluation results of ecological structure of waterfront plant landscape in Xingqing Palace Park, Xi’an.

Number	Evaluation Value	Comprehensive Evaluation Index	Evaluation Grade	Number	Evaluation Value	Comprehensive Evaluation Index	Evaluation Grade
S1	2.99	64.75%	Preferably	S17	2.54	55.10%	Moderate
S2	3.51	76.08%	Preferably	S18	2.96	64.19%	Preferably
S3	2.73	59.11%	Moderate	S19	2.38	51.57%	Moderate
S4	3.34	72.34%	Preferably	S20	4.02	87.15%	High quality
S5	4.22	91.38%	High quality	S21	2.95	63.84%	Preferably
S6	3.83	82.96%	High quality	S22	3.32	72.01%	Preferably
S7	3.50	75.93%	Preferably	S23	2.68	58.05%	Moderate
S8	3.62	78.45%	Preferably	S24	2.55	55.33%	Moderate
S9	3.17	68.63%	Preferably	S25	3.20	69.41%	Preferably
S10	3.30	71.61%	Preferably	S26	3.35	72.63%	Preferably
S11	3.67	79.57%	Preferably	S27	2.50	54.20%	Moderate
S12	4.32	93.73%	High quality	S28	3.34	72.43%	Preferably
S13	3.91	84.74%	High quality	S29	2.67	57.96%	Moderate
S14	2.83	61.25%	Preferably	S30	3.23	69.98%	Preferably
S15	3.58	77.62%	Preferably	S31	3.67	79.52%	Preferably
S16	2.71	58.73%	Moderate	S32	2.73	59.11%	Moderate

This result shows that ecology has the greatest impact on atmosphere in plant landscapes, but aesthetic elements and cultural elements among human factors are also important. First of all, in the process of creating a garden atmosphere, landscape designers should meet the needs of “green” and “sustainable” ecological development of the landscape in the “ecological structure”, pay attention to species diversity and ecological stability, and let plants show their vitality. The regional character of the plant communities, healthy water ecology, and biodiversity form a healthy ecosystem that sets the atmospheric tone of the waterfront plant landscape. Under the ecological environment of sustainable development, plants grow luxuriantly and vigorously, while nature itself contains vigorous vitality, and the garden presents a positive emotional atmosphere, such as comfort, cosiness and pleasure; under the environment of ecological imbalance, plants grow and develop poorly, thus people separate from natural environment, and the garden presents a negative emotional atmosphere, such as decadence, depression and sadness. “A thing is correct only when it helps to protect the harmony, stability and beauty of the biological community; otherwise, it is wrong.” [54] In other words, it is beautiful to have a harmonious and stable ecological community. In addition, Xingqing Palace Park is designed in the Chinese Tang Dynasty garden design style, so the atmosphere and style need to highlight the “prosperous Tang Dynasty atmosphere”, which is a strong national self-confidence and high-spirited spiritual outlook. Therefore, in the process of planning Xingqing Palace Park, designers should fully consider the ecological elements of the plant community, establish an ecologically healthy waterfront space, and create a positive, confident, and prosperous waterfront space on the basis of maintaining clear water and ecological integration of plant landscapes. Atmosphere, giving visitors positive emotional feedback.

Secondly, designers need to focus on the harmony between mountains, waters, plants and buildings in order to realize the aesthetics of the planted landscape. Hegel said in “*Aesthetics*”: “Garden art not only creates an environment for the spirit, a second nature, and uses a completely new way to build it from the beginning, but also incorporates natural scenery into the composition design of the building… …and the real garden art… is a kind of painting that keeps natural things in their natural form and strives to imitate free nature. It gathers together all the things that can make people feel relaxed and happy in the natural scenery to form a whole, such as rocks and their rough natural volumes, valleys, woods, lawns, winding streams, large rivers with lively atmosphere on their banks, calm lakes with flowers and trees, cascading waterfalls, etc. Chinese Garden art has long incorporated the entire natural scenery, including lakes, islands, rivers, rockeries, vistas, etc. into the garden [55]”. The beauty of the garden is a comprehensive beauty that integrates ecology, architecture and art, and its essence is the harmony between man and nature [56]. The tradition of harmony continues in Xingqing Palace Park, where there are many natural objects in the architectural space, and when people enter the space, they are enveloped by the atmosphere created by plants, artistic configurations, architecture, and landscapes. Through its form, color, smell, sound and other extensions and symbolism, nature evokes human emotions and converses with human beings. The extension and form of natural existences is what Böhme calls “the ecstasy of things”. People perceive and feel through the “ecstasy of things”, and then these entities come to the aesthetics of atmosphere [57]. The natural elements that become aesthetic objects are able to connect matter and consciousness through the overlap of time and space, connecting the individual and the whole, the finite and the infinite, and thus stimulating a variety of emotions.

Finally, in the selection of plants, the designer attaches importance to the cultural connotation and cultural characteristics of plants, and uses the cultural connotation of plants to give the landscape a unique atmosphere. Culture, as a broad extension of things, exists in a quasi-objective, i.e., more fixed form. When people enter the plant landscape, they will be surrounded by the atmosphere constituted by plant culture, and immersed in the cultural context, the subject’s mind and the cultural connotation of plants to achieve a harmony. In the long process of development of history, willow, reed and lotus have rich cultural meaning due to literary writing. Take lotus as an example, lotus is honored as a sacred object of Buddhism, Taoism and Confucianism, with the symbol of immortality, elegance and purity, and a gentleman among flowers. When visitors are in the lotus landscape, they are surrounded by an elegant atmosphere. Their hearts rise with respect for all things in the universe, and they feel that their body and mind absorb the longevity energy of the lotus. Because of the lotus landscape in Xingqing Park, many tourists come here to practice Tai Chi, take photos, and visit. Xingqing Palace Park has witnessed the rise and fall of history. After several declines and dilapidations, it finally evolved from a royal garden into a free park for civilian entertainment. During the evolution of history, the plants in Xingqing Palace have brought with them the cultural meaning of the impermanence of life and the changing of things and people. Through the cultural meaning of plants, visitors can trigger their own thoughts and perceptions about the universe, history, and life. Their perceptions transcend reality and span thousands of years of history, from a prosperous and magnificent atmosphere to a tragic atmosphere of historical changes. The extension of culture, objects, physical presence, and spiritual enlightenment expansion, the physical and mental experience and the garden atmosphere are integrated into a complex aesthetic experience.

### 4.2 Correlation analysis between SBE and AHP evaluation results

Kendall’s W method was used to conduct a correlation test on the evaluation results of SBE and AHP (Table 9). The results show that w = 0.901, the asymptotic significance is P = 0.004<0.01, and the probability guarantee level is 99%. The ranking results of AHP and SBE evaluation have high consistency. And the final evaluation results of SBE and AHP are obtained.

**Table 9:** Test results of Kendall coefficient of concordance.

Case number	Kendall’s W	Test Statistic	Degrees of Freedom	Asymptotic Sig.
2	0.943	58.463	31	0.002

Table 10. Final results of 2 evaluation methods of ecological structure of waterfront plant landscape in Xingqing Palace Park, Xi’an

**Table 10:** AHP Evaluation results of ecological structure of waterfront plant landscape in Xingqing Palace Park, Xi’an.

Sample number	AHP scores	AHP Standardized score	AHP order	SBE scores	SBE Standardized score	SBE order	Total score	Total order
S1	2.99	-0.46	20	17.5	-0.31	19	-0.77	21
S2	3.51	0.54	10	18.35	0.52	10	1.06	10
S3	2.73	-0.96	25	17.1	-0.7	25	-1.66	25
S4	3.34	0.21	14	18.25	0.42	14	0.63	14
S5	4.22	1.90	2	19.6	1.74	2	3.64	2
S6	3.83	1.15	5	18.85	1.01	5	2.16	4
S7	3.50	0.52	11	18.25	0.42	14	0.94	11
S8	3.62	0.75	8	18.35	0.52	10	1.27	9
S9	3.17	-0.11	19	17.25	-0.55	24	-0.66	19
S10	3.30	0.14	16	17.4	-0.41	21	-0.27	16
S11	3.67	0.85	7	18.9	1.06	4	1.91	6
S12	4.32	2.09	1	20.05	2.18	1	4.27	1
S13	3.91	1.31	4	19.05	1.2	3	2.51	3
S14	2.83	-0.76	23	18.25	0.42	14	-0.34	17
S15	3.58	0.67	9	18.7	0.86	7	1.53	8
S16	2.71	-0.99	26	16.65	-1.14	28	-2.13	27
S17	2.54	-1.32	30	17.3	-0.5	23	-1.82	26
S18	2.96	-0.52	21	17.65	-0.16	17	-0.68	20
S19	2.38	-1.63	32	16.6	-1.19	29	-2.82	31
S20	4.02	1.52	3	18.3	0.47	12	1.99	5
S21	2.95	-0.53	22	17.45	-0.36	20	-0.89	22
S22	3.32	0.17	15	18.55	0.71	8	0.88	12
S23	2.68	-1.05	27	16.3	-1.48	31	-2.53	30
S24	2.55	-1.30	29	17.6	-0.21	18	-1.51	24
S25	3.20	-0.06	18	16.8	-0.99	26	-1.05	23
S26	3.35	0.23	12	18.3	0.47	12	0.7	13
S27	2.50	-1.40	31	15.4	-2.36	32	-3.76	32
S28	3.34	0.21	14	18.1	0.28	16	0.49	15
S29	2.67	-1.07	28	16.65	-1.14	28	-2.21	29
S30	3.23	0.00	17	17.35	-0.46	22	-0.46	18
S31	3.67	0.85	7	18.75	0.91	6	1.76	7
S32	2.73	-0.96	25	16.55	-1.24	30	-2.2	28

**Fig 1:**
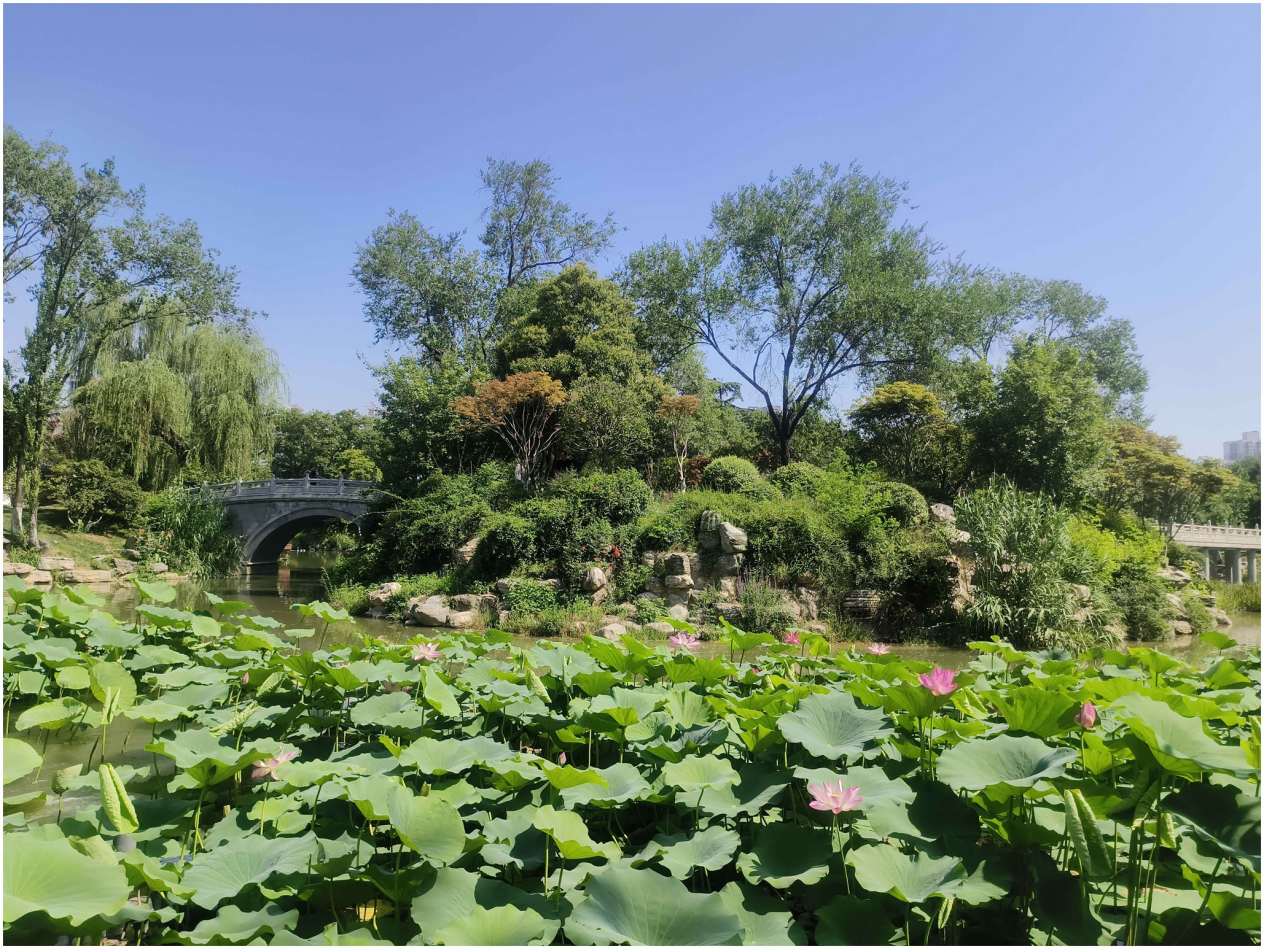
No.5 waterfront plant landscape.

As can be seen from Table 10, most of the evaluation results of the 2 methods tend to be consistent, and a few of the evaluation results are quite different. Analyzing the changes in the polyline in Fig 1 shows that the fluctuations in the AHP and SBE evaluation score curves are relatively similar, which further illustrates that the ecological structure affects the landscape atmosphere and thus affects the aesthetic expression of the landscape. For example, the rank of SBE, AHP and comprehensive evaluation results of No.5 waterfront plant landscape are all 2, and the evaluation results are basically consistent. No.5 waterfront plant landscape (Fig 1) on the basis of the original terrain, planting five cornered maple, ligustrum lucidum, heather, small-leaf boxwood, chinese white poplar, iris, calamus, hibiscus, elm, lotus, etc, has formed the landscape effect of lush greenery, hierarchical, and flowers competing for color, which focuses on creating the atmosphere of full of vitality and prosperity. Lotus, calamus and other plants have unique cultural connotations in Chinese culture, implying beautiful life and love, while giving people a pleasant atmosphere, generating a joyful atmosphere with the help of rich colors and symbols.

**Fig 2:**
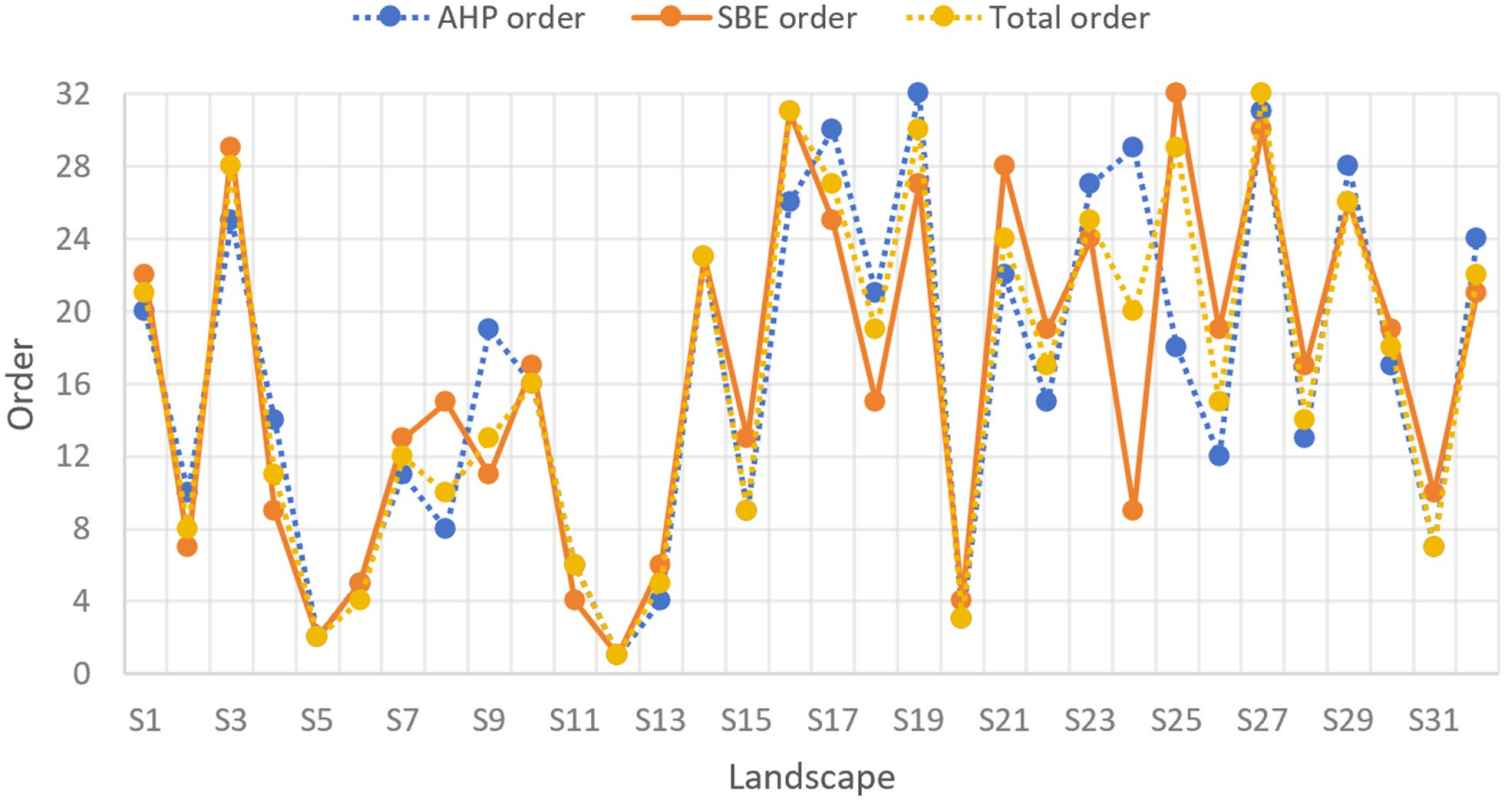
Comparison of the evaluation results of the two methods.

**Fig 3:**
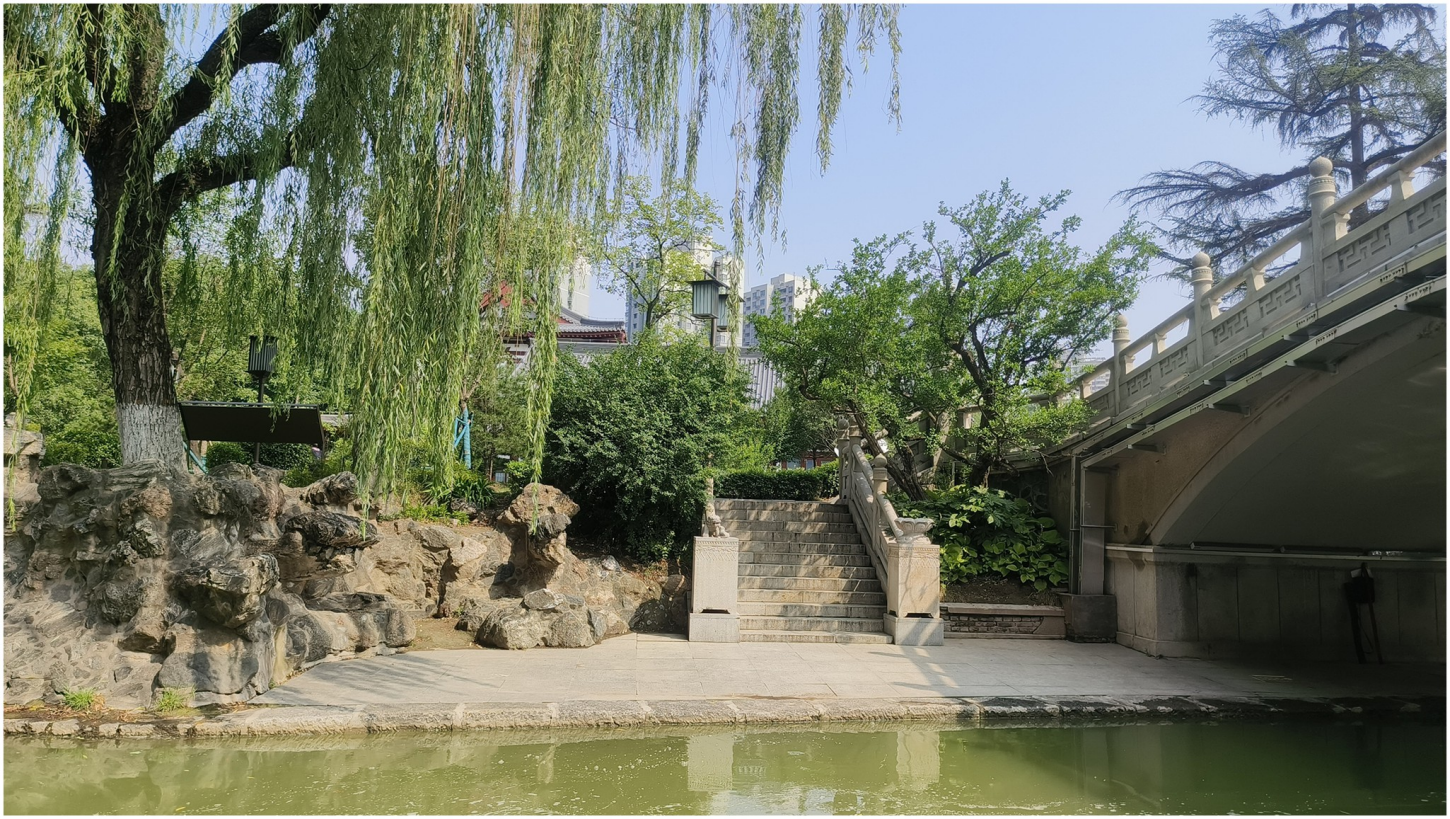
No.25 waterfront plant landscape.

Therefore, the results of both evaluation methods are at the top. According to the difference between the two evaluation results (Fig 2), it can be found that the difference between sample plots S24 and S25 is more prominent. For example, No.25 waterfront plant landscape (Fig 3), the rank of AHP is 18, while the rank of SBE is 26. The evaluation result of AHP is higher, and there is a significant difference between the two. The waterfront plant landscape of No. 25 is dominated by willows, with hostas, boxwoods, white poplars, spring orchids and other plants. From a quantitative perspective, there are 5 plant species here, with high visual hierarchy and regional plant communities. Among plants The artistic configuration includes arch bridges, rockeries, stone stairs and other artistic configurations. Compared with other waterfront plants, there are more ecological structural elements to create an atmosphere, so the AHP score is higher. However, from an intuitive point of view, the overall number of plants on this hydrophilic platform is scarce, so it is impossible to create a spatial atmosphere. Moreover, the color of the water surface in the picture is yellow-green with bubbles. The viewer can clearly see that the water body is seriously eutrophic, and the harmonious and leisurely atmosphere is destroyed. The atmosphere is ruined and therefore the SBE score is lower.

**Fig 4:**
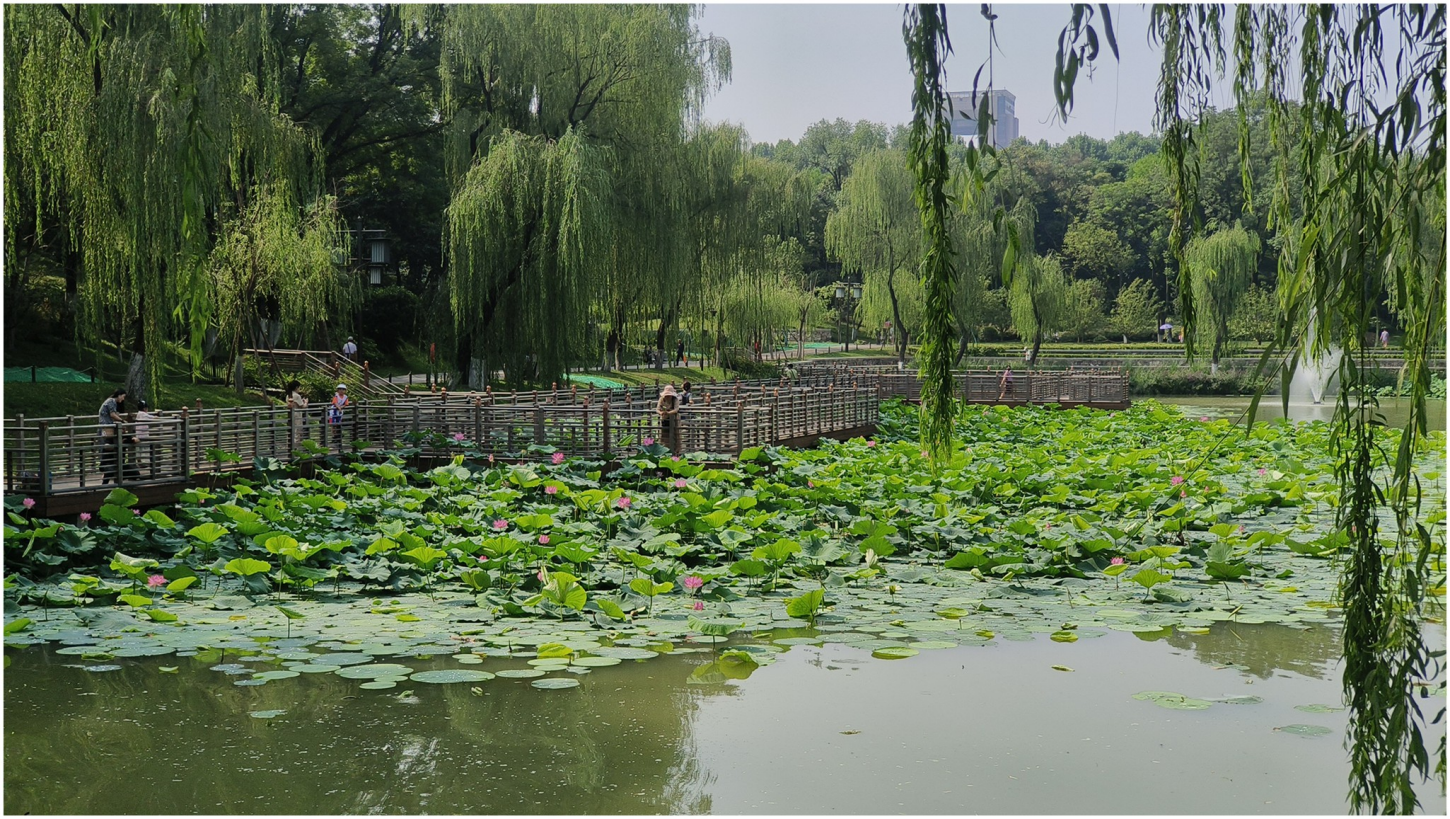
No.24 waterfront plant landscape.

For example, No. 24 waterfront plant landscape (Fig 4), the rank of AHP is 29, while the rank of SBE is 18. The evaluation result of SBE is higher, and there is a significant difference between the two. The main plant species in No. 24’s waterfront plant landscape is lotus, so there is only one species diversity in the quantitative indicators. The visual hierarchy of plants is single. Each lotus flower bud blooms for only 2–3 days, and the overall plant viewing period is only about 20 days, so the AHP evaluation result is low. However, although this waterfront plant landscape has a single type of plants, its colors are fresh and neat. At the same time, it echoes the surrounding covered bridges, arch bridges, weeping willows, etc., creating a “white bird and red lotus attracting the painted rafters, and the red bridge can be seen in the shadow of the weeping willow.” The artistic conception is beautiful and the lotus has rich cultural connotations, forming a tranquil and elegant atmosphere. Overall, AHP generally divides the elements that create atmosphere in the evaluation process and does not fully pay attention to tourists’ feelings about the overall landscape; while SBE focuses on users’ intuitive perception of plant landscape units and cannot refine the components of atmosphere. By combining the two, the comprehensive evaluation results obtained effectively make up for the shortcomings of using AHP or SBE alone, making the evaluation process take into account both expert considerations and user preferences, and adapting to the objectivity and subjectivity of atmosphere evaluation to a certain extent.

## 5 Conclusions

In the theory of environmental aesthetics, atmosphere is the primary factor that affects the natural beauty and subjective perception of gardens. Therefore, it is of great significance to discover the factors that affect the atmosphere and then transform the garden atmosphere to improve the beauty of the landscape. This study traces the divergence of atmosphere back to ecological structure, subdivides the components of ecological structure, and analyzes the impact of ecological structure on landscape aesthetic atmosphere. Therefore, this paper constructs an evaluation index system for the ecological structure of waterfront plant landscapes in Xi’an Xingqinggong Park, uses SBE-AHP to measure the scoring index of 32 landscape units, and establishes the connection between the weight of the ecological structure components and the beauty of the landscape.

First, the impact path between ecological structure, atmosphere perception, and aesthetic preferences is established using principal component analysis. Various elements in the ecological structure affect people’s psychological factors such as perception, directness, sensation, rationality, and emotion. These psychological factors integrate instantaneously to form a judgment of the atmosphere, which ultimately affects an individual’s aesthetic preferences for the landscape.

Secondly, ecological health affects the expression of landscape beauty and atmosphere. Ecology remains the most important factor in people’s aesthetic experiences. People do not directly appreciate ecological diversity, plant species, or plant characteristics, but these elements act together in a vague and holistic manner on people’s aesthetic judgments, which is the unity of truth, goodness, and beauty. Although there are many elements affecting the atmosphere of the garden plant landscape, such as artistic configuration, plant shape, and precious proportion, it is still the ecosystem that is the core element affecting the atmosphere as an expression of the wholeness of the plant landscape. Sustainable ecosystems create a species-rich, healthy, bright and pleasant atmosphere, which in turn affects the physical presence of visitors, and inter-subjectively matches the exuberant vitality of plants.

Thirdly, aesthetic and cultural element enrich the breadth of plants, extending the physical plant into a non-physical, infinite, imaginative, emotional atmosphere. Specifically, plants with aroma trigger the sense of smell, then process the odor information through the tonsils thereby causing an emotional response, and finally evoke memories. The designer selects native plants that match the theme culture of the garden and plants with positive cultural meanings, and displays the harmony between water and plants in the treatment of space, allowing tourists to visit in a comfortable atmosphere. Within the same category of riparian plants, those with positive cultural implications are more popular.

Finally, when designing specific applications, it should also be “tailored to local conditions” and choose plants that are suitable for the local geographical environment, rather than forcibly transplanting plants that are not suitable for the area solely for the purpose of ecological diversity. This not only increases design costs but also disrupts ecological harmony by using unsuitable plants. Atmosphere is a conceptual idea based on emotion, and while quantification can help find design ideas that provide a better experience for most people, it can also lead to the homogenization of landscape riparian design templates in the future.

However, there are still some limitations in the current research. Firstly, the discussion on core concepts such as ecological structure and atmosphere is relatively superficial, failing to deeply explore the logic and connections between them. Secondly, in examining the pathway by which ecological structures influence atmosphere, this study primarily focuses on psychological and philosophical analysis, lacking sufficient on-site questionnaires and interviews to describe the aesthetic atmosphere of landscapes. This gap prevents us from exploring specific aesthetic atmospheres such as relaxation, tension, decadence, and liveliness. These limitations, however, provide valuable insights for further research.

## Supporting information

S1 Fig Sample landscape photos. Own photographs.
(PDF)

S1 File AHP evaluation results. (XLSX)

S2 File Summary of results from the Delphi expert survey method. (XLS)

S3 File Original data of Scenic Beauty Estimation (SBE) scores of 100 samples. (XLSX)

S4 File Evaluation index system of ecological structure of waterfront plant landscape in Xingqinggong Park, Xi’an. (PDF)
