# Supplementary material for: How the ecological structure affects the aesthetic atmosphere of the landscape: Evaluation of the landscape Beauty of Xingqing Palace Park in Xi’an
Source: PLoS One. 2024 May 15;19(5):e0302855. doi: 10.1371/journal.pone.0302855 (PMC11095750; doi:10.1371/journal.pone.0302855)
Supplement: S1 Fig — Own photographs. (PDF) [file pone.0302855.s001.pdf]

Sample landscape photos

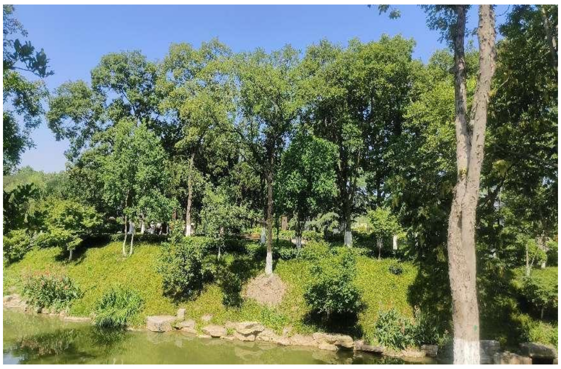
NO.1 a

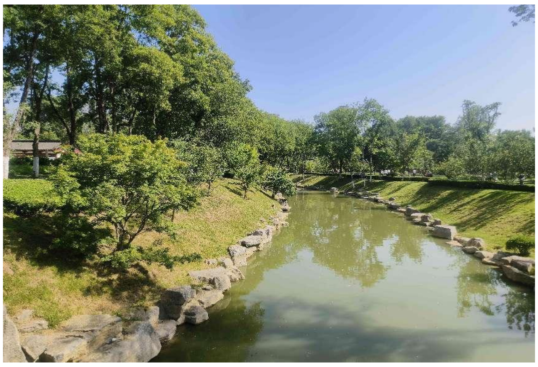
NO.1 b

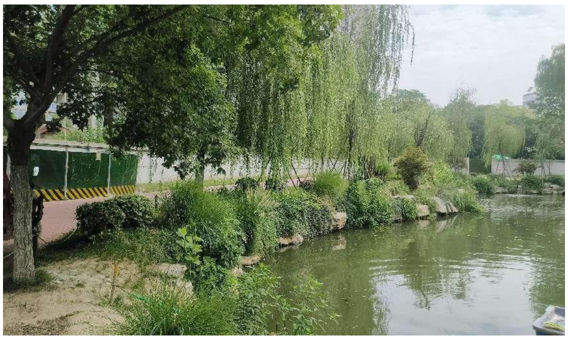
NO.2 a

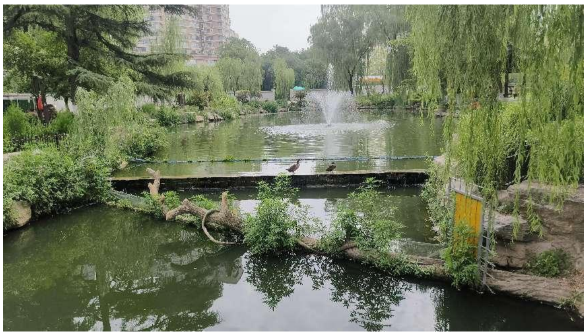
NO.2 b

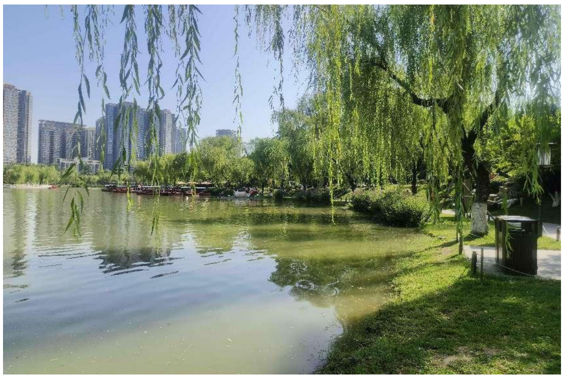
NO.3 a

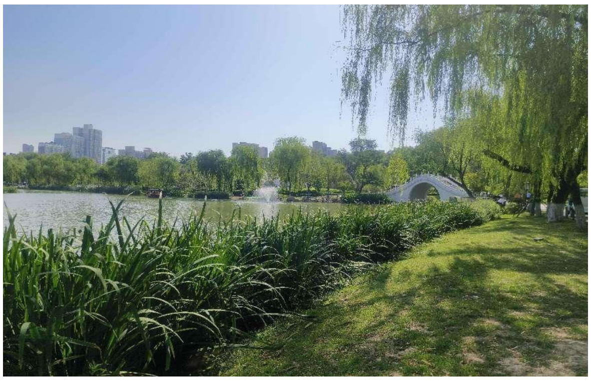
NO.3 b

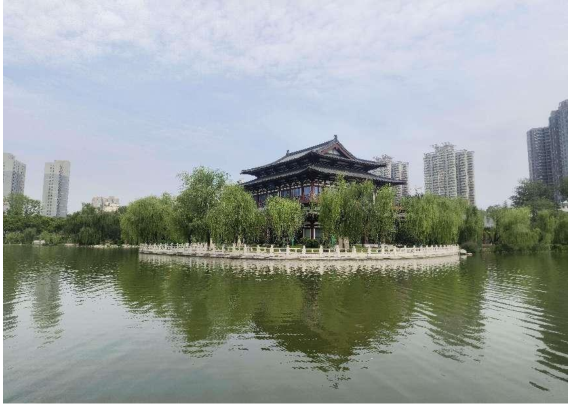
NO.4 a

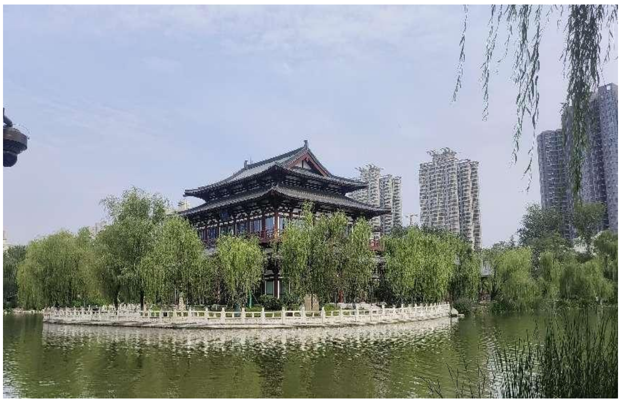
NO.4 b

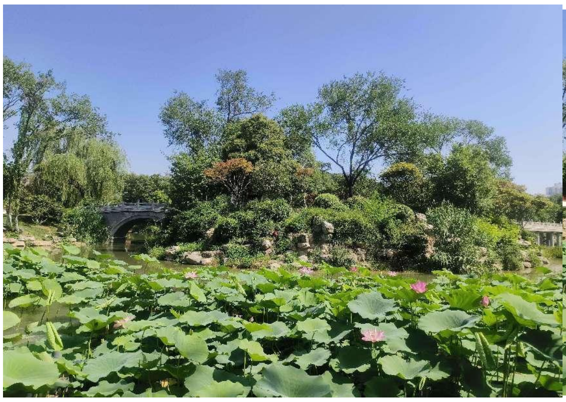
NO. 5 a
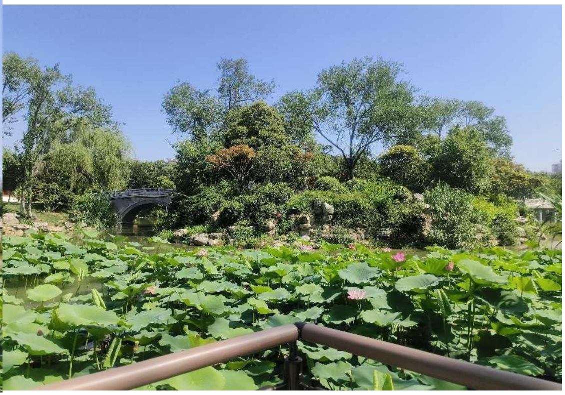
NO.5 b
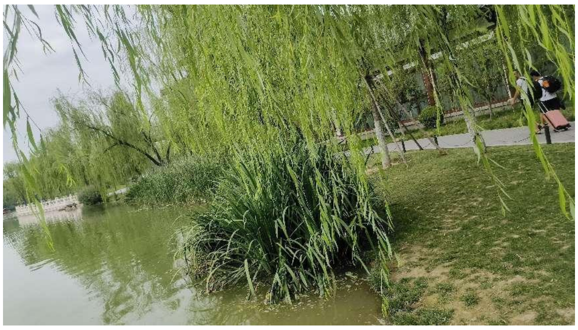
NO.6 a
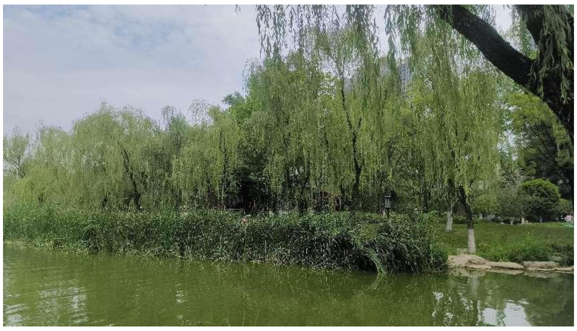
NO.6 b
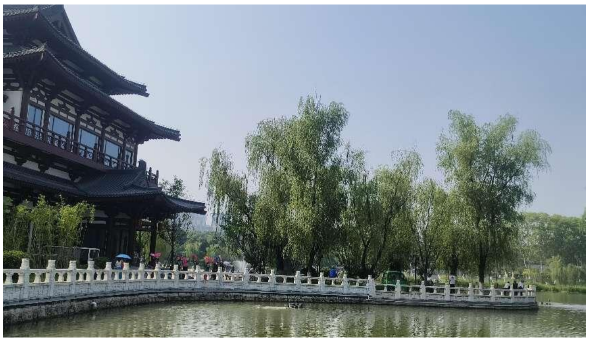
NO.7 a
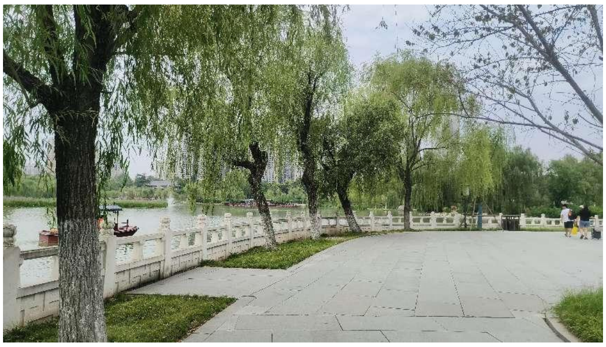
NO.7 b
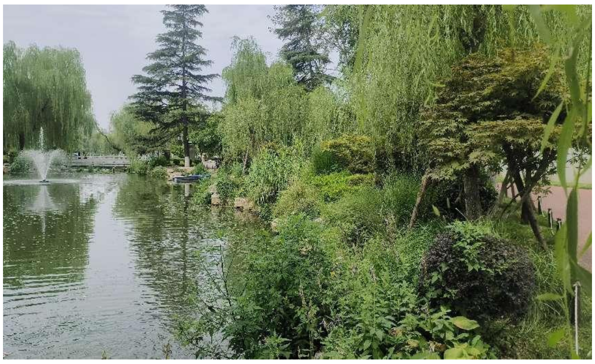
NO.8a
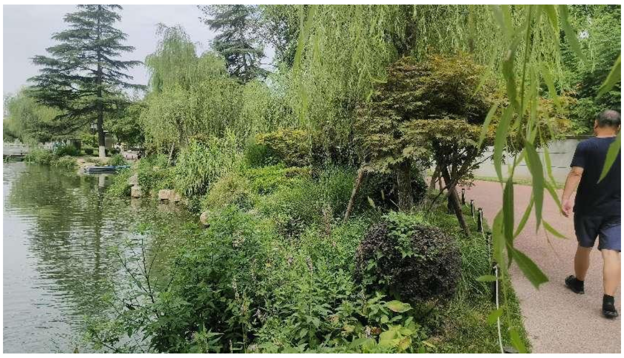
NO.8 b

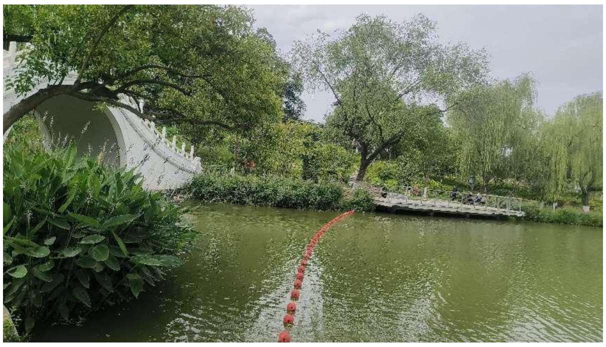

NO.9 a

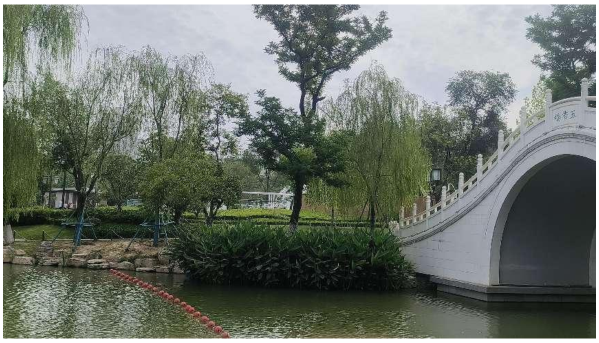

NO.9 b

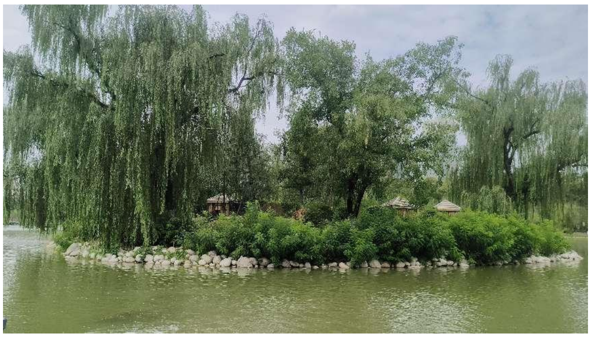

NO. 10 a

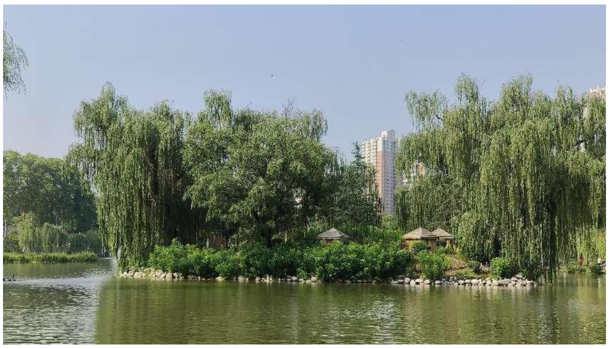

NO.10 b

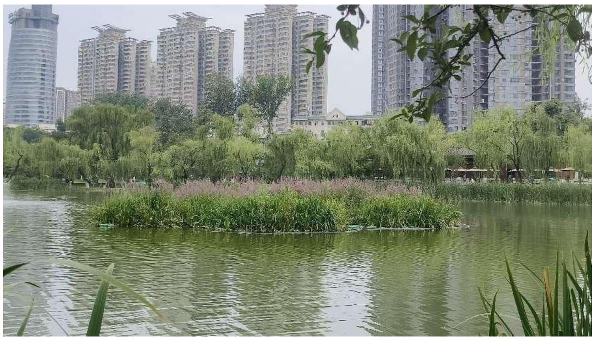

NO.11 a

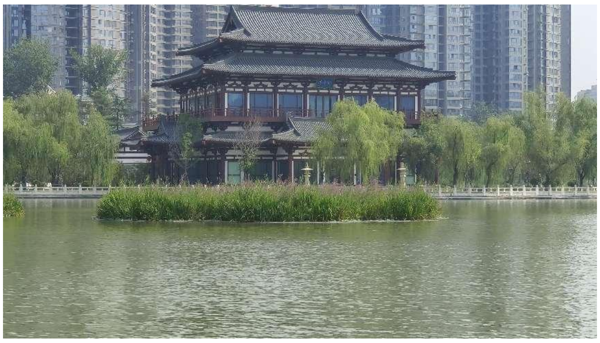

NO.11 b

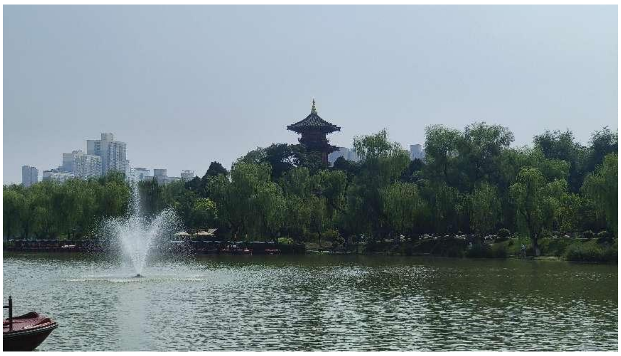

NO. 12 a

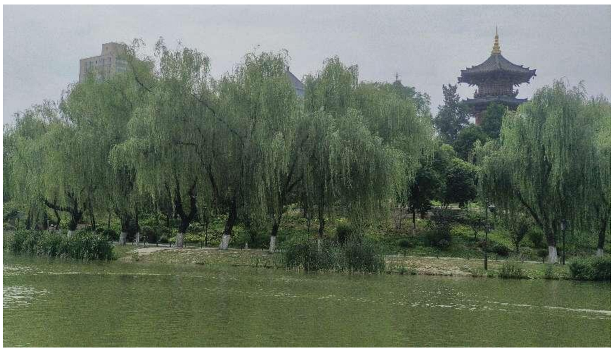

NO. 12b

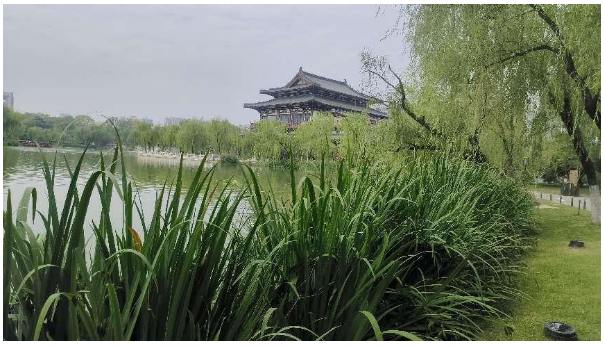

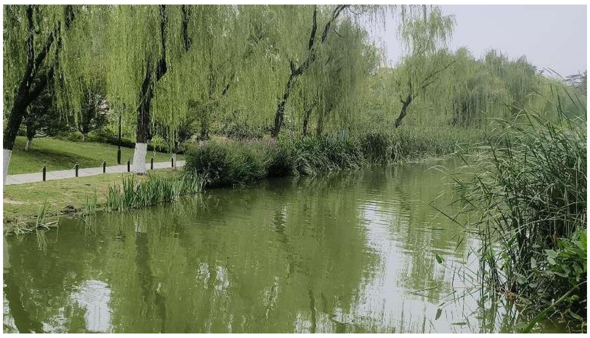

NO.13 a

NO. 13 b

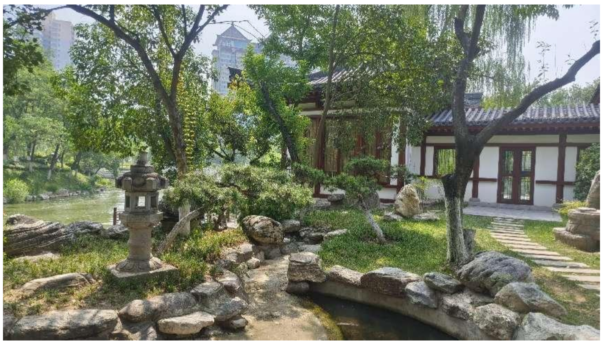

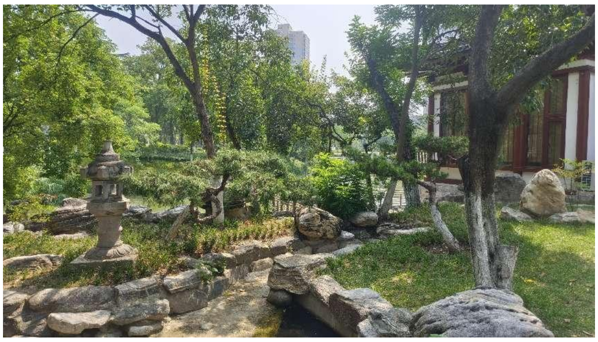

NO.14 a

NO.14 b

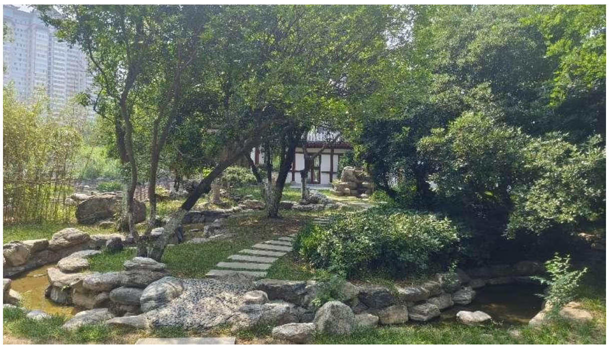

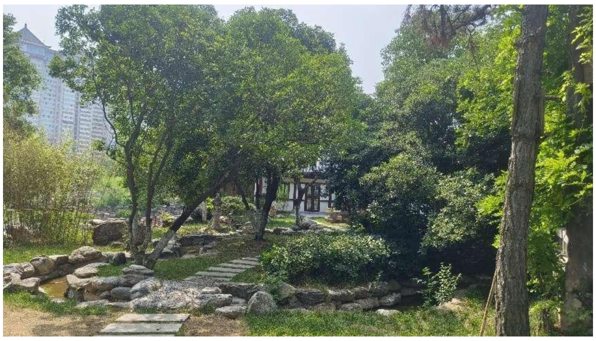

NO.15 a

NO.15 b

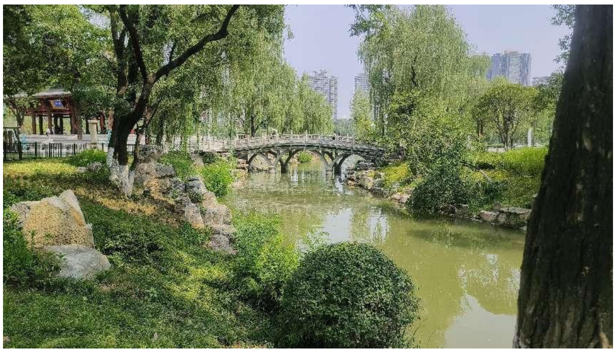

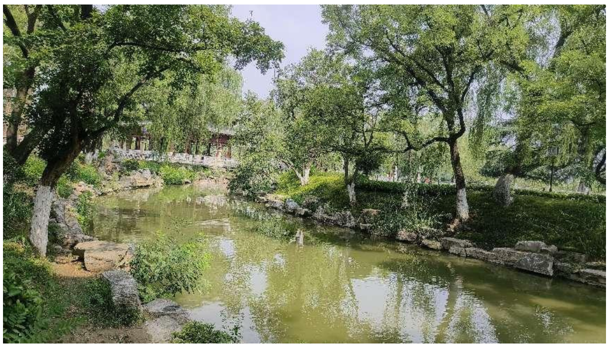

NO.16 a

NO.16 b

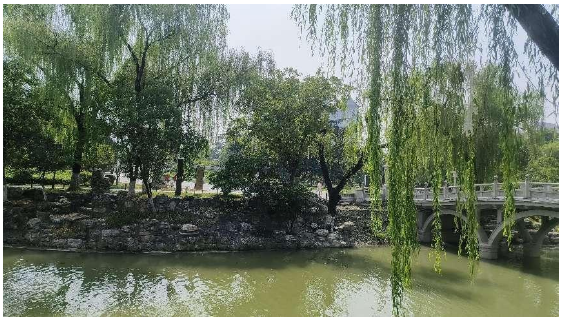
NO.17 a
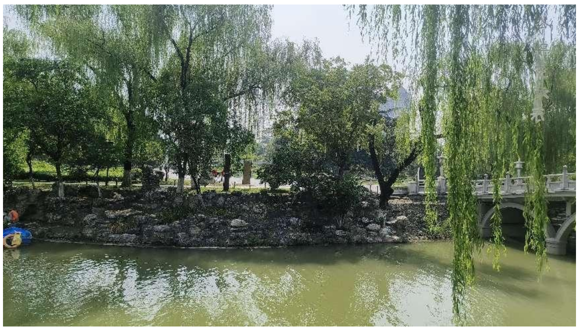
NO.17 b
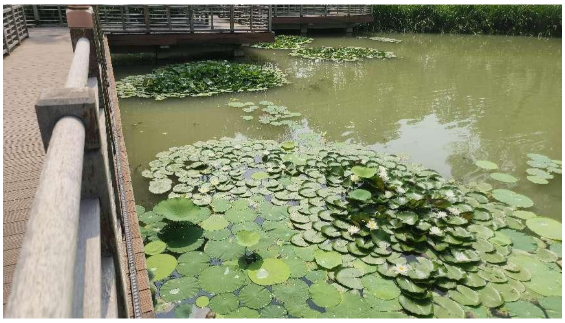
NO.18 a
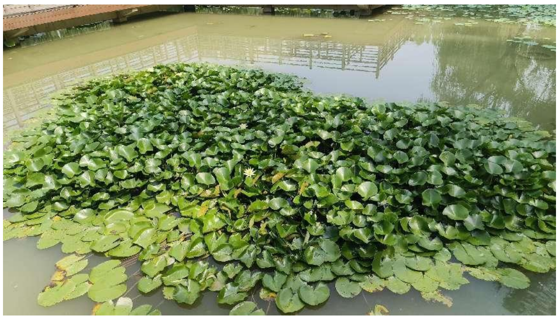
NO.18 b
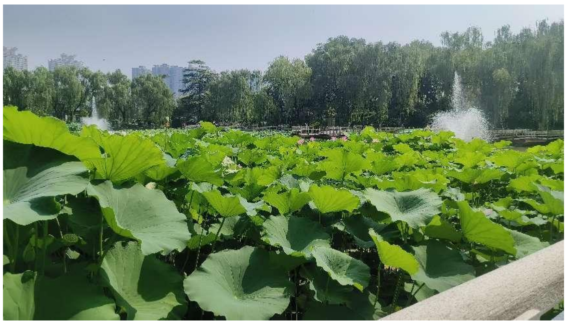
NO.19 a
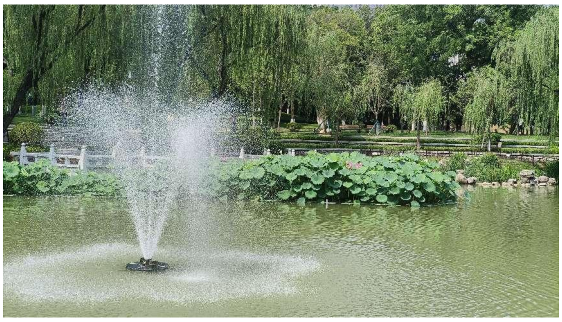
NO.19 b
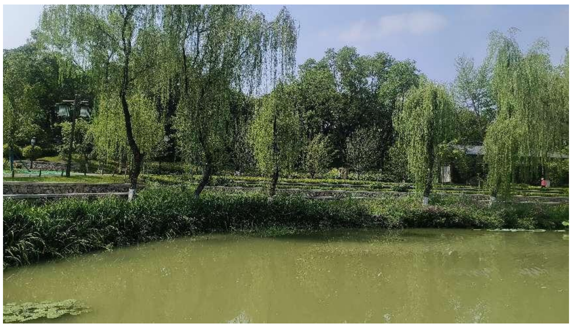
NO.20 a
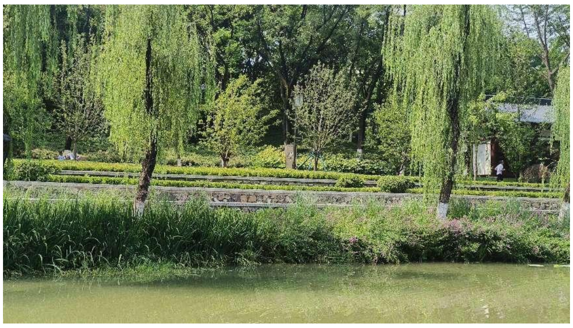
NO.20 b

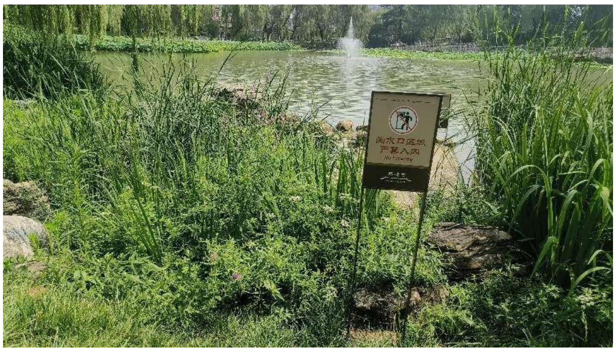

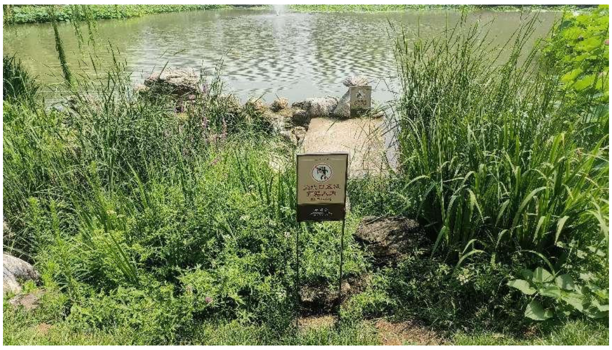

NO.21 a

NO.21b

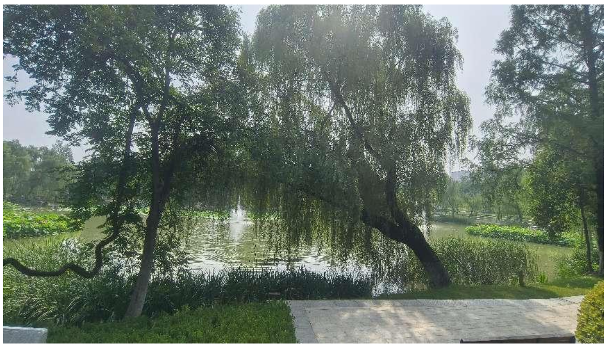

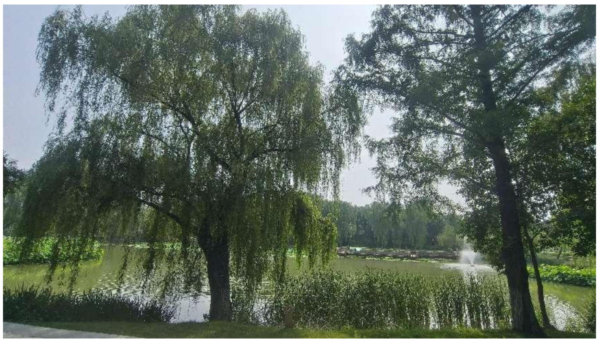

NO.22 a

NO.22 b

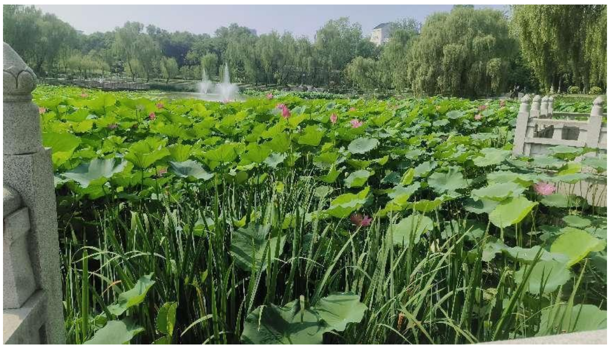

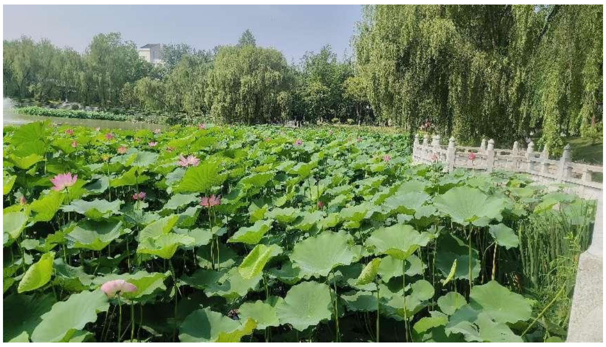

NO.23 a

NO.23 b

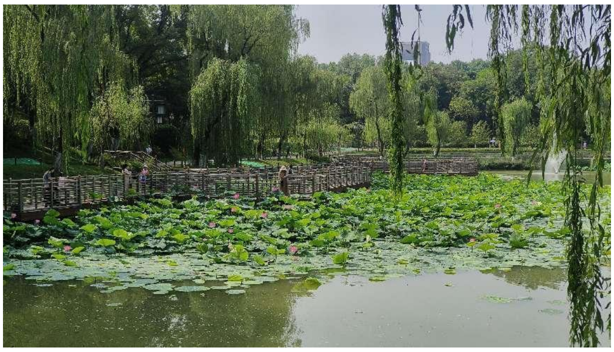

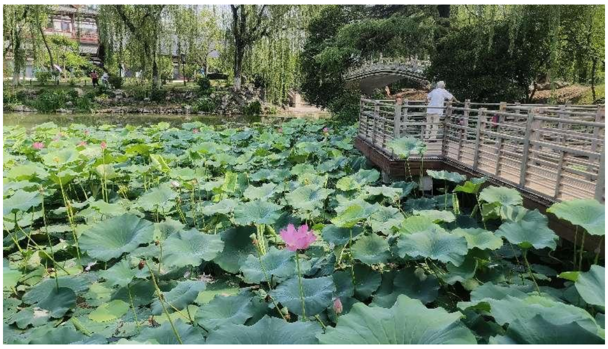

NO.24 a

NO.24 b

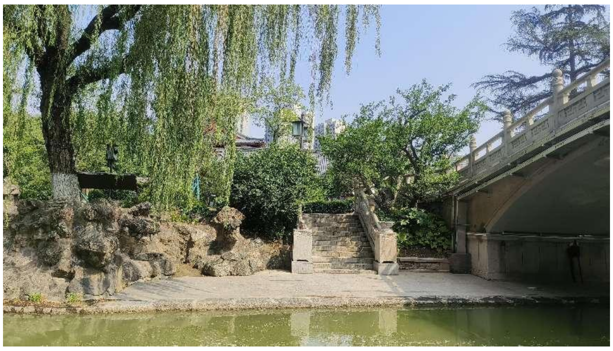
NO.25 a

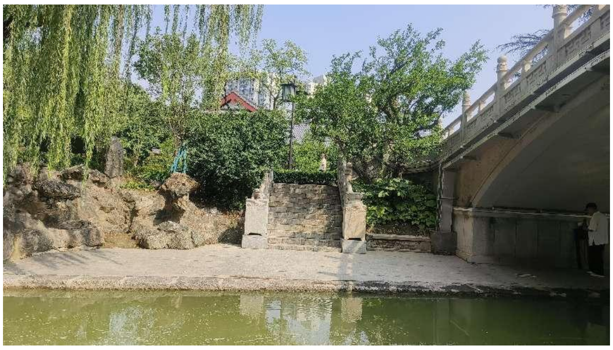
NO.25 b

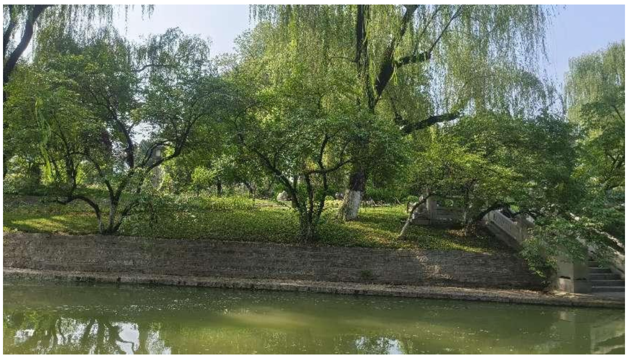
NO.26 a

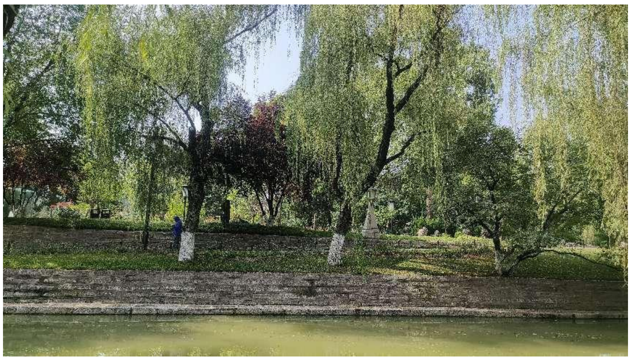
NO.26 b

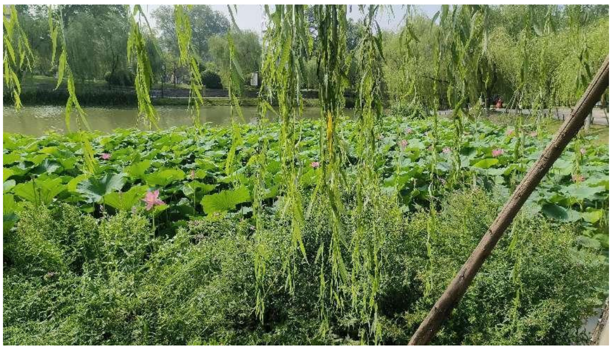
NO.27 a

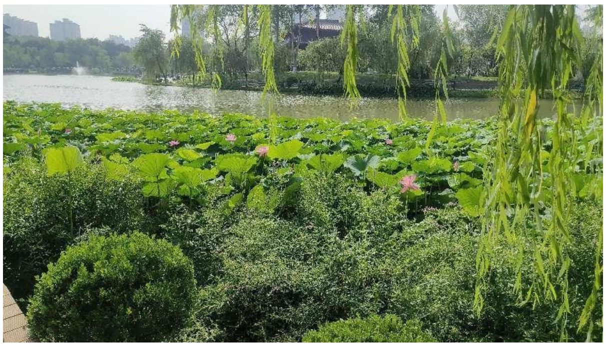
NO.27 b

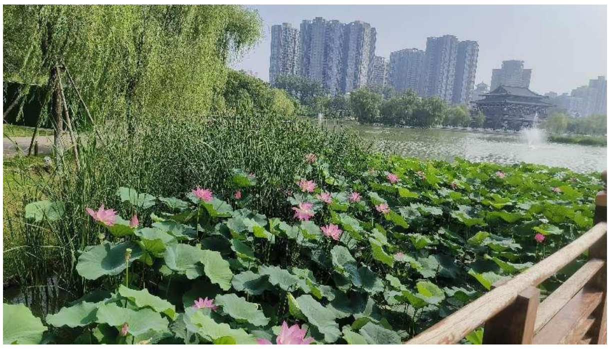
NO.28 a

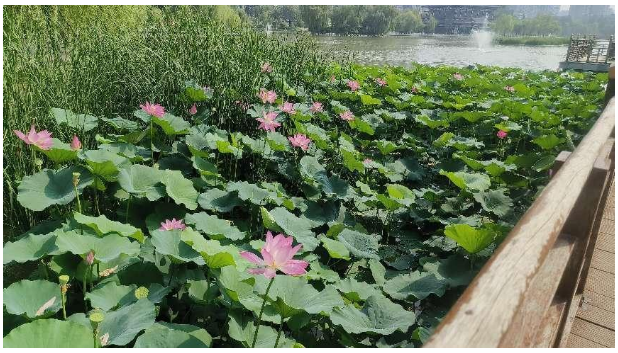
NO.28 b

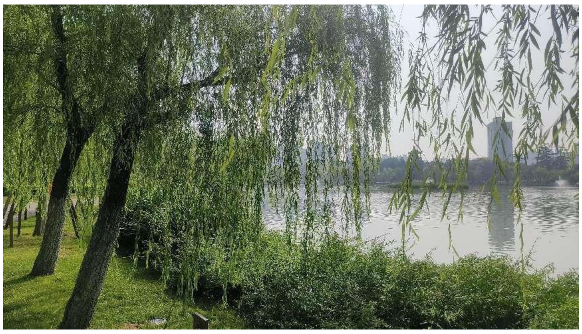

NO. 29 a

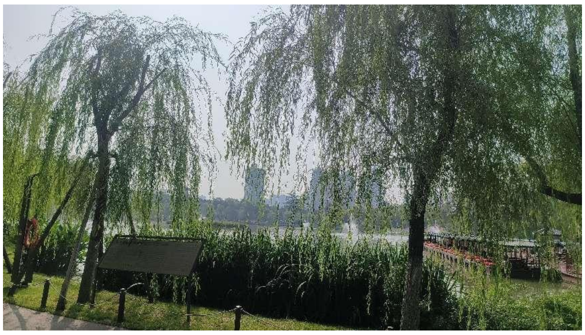

NO.29 b

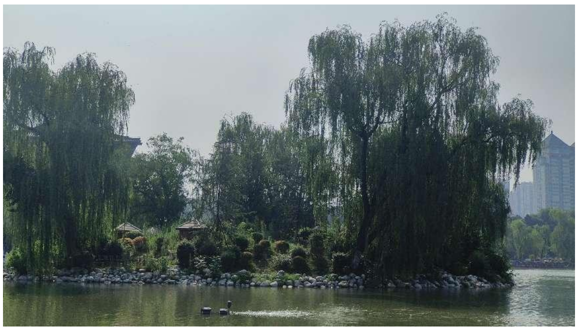

NO.30 a

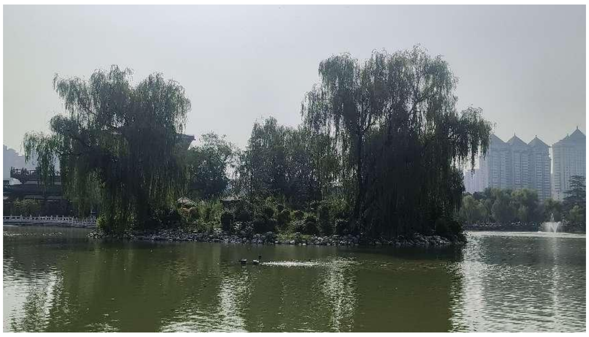

NO.30 b

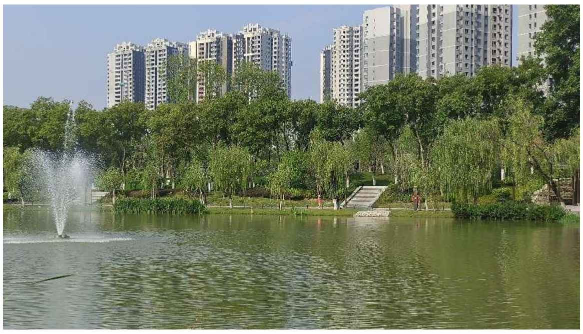

NO.31 a

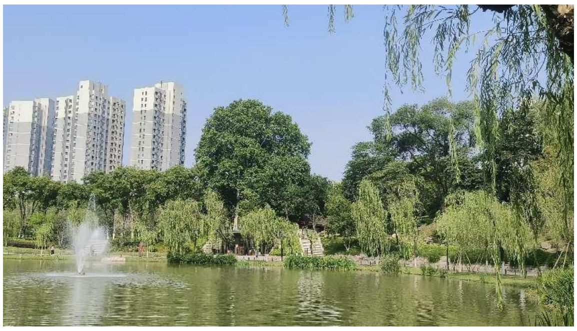

NO.31 b

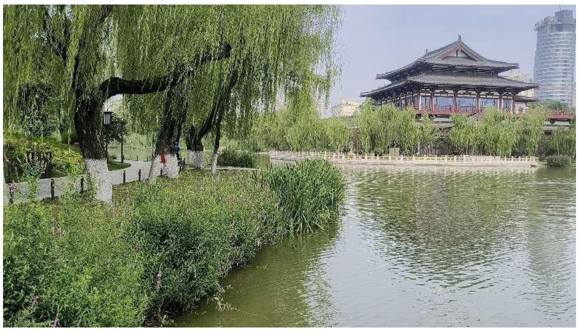

NO.32 a

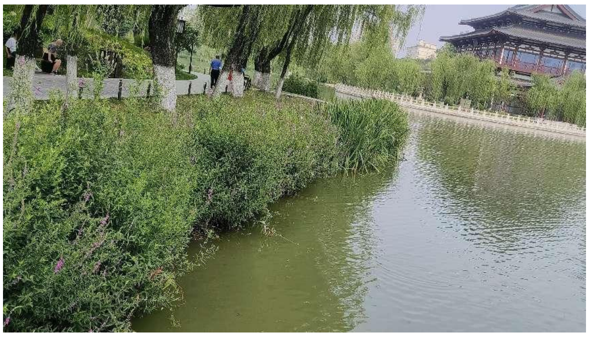

NO.32 b
