# Supplementary material for: How the ecological structure affects the aesthetic atmosphere of the landscape: Evaluation of the landscape Beauty of Xingqing Palace Park in Xi’an
Source: PLoS One. 2024 May 15;19(5):e0302855. doi: 10.1371/journal.pone.0302855 (PMC11095750; doi:10.1371/journal.pone.0302855)
Supplement: S4 File — (PDF) [file pone.0302855.s005.pdf]

Evaluation index system of ecological structure of waterfront plant landscape in Xingqinggong Park, Xi'an

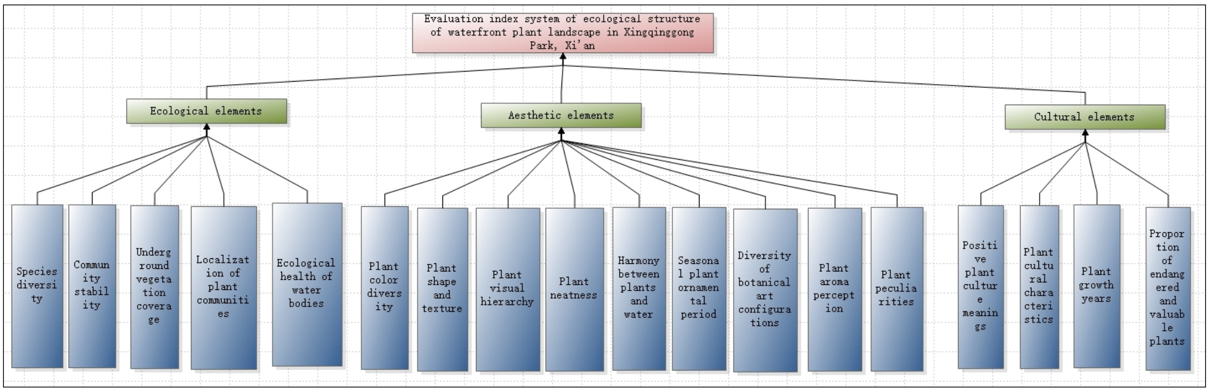

标度类型:1-9

方案层中要素对决策目标的排序权重

备选方案	权重
Localization of plant communities	0.2189
Ecological health of water bodies	0.1463
Species diversity	0.1106
Positive plant culture meanings	0.0887
Community stability	0.0777
Harmony between plants and water	0.0653
Plant cultural characteristics	0.0467
Plant aroma perception	0.0455
Underground vegetation coverage	0.0401
Seasonal plant ornamental period	0.0344
Diversity of botanical art configurations	0.0295
Plant shape and texture	0.0252
Plant visual hierarchy	0.0185
Plant color diversity	0.0133
Plant growth years	0.0131
Plant neatness	0.0110
Proportion of endangered and valuable plants	0.0086
Plant peculiarities	0.0065

第1个中间层中要素对决策目标的排序权重

中间层要素	权重
Ecological elements	0.5936
Aesthetic elements	0.2493
Cultural elements	0.1571

1. Evaluation index system of ecological structure of waterfront plant landscape in Xingqinggong Park, Xi'an 一致性比例：0.0516；对"Evaluation index system of ecological structure of waterfront plant landscape in Xingqinggong Park, Xi'an"的权重：1.0000；λmax：3.0536

Evaluation index system of ecological structure of waterfront plant landscape in Xingqinggong Park, Xi'an	Ecological elements	Cultural elements	Aesthetic elements	Wi
Ecological elements	1.0000	3.0000	3.0000	0.5936
Cultural elements	0.3333	1.0000	0.5000	0.1571
Aesthetic elements	0.3333	2.0000	1.0000	0.2493

2. Ecological elements 一致性比例：0.0321；对"Evaluation index system of ecological structure of waterfront plant landscape in Xingqinggong Park, Xi'an"的权重：0.5936；λmax：5.1439

Ecological elements	Species diversity	Community stability	Underground vegetation coverage	Localization of plant communities	Ecological health of water bodies	Wi
Species diversity	1.0000	2.0000	3.0000	0.5000	0.5000	0.1863
Community stability	0.5000	1.0000	3.0000	0.3333	0.5000	0.1308
Underground vegetation coverage	0.3333	0.3333	1.0000	0.2500	0.3333	0.0676
Localization of plant communities	2.0000	3.0000	4.0000	1.0000	2.0000	0.3688
Ecological health of water bodies	2.0000	2.0000	3.0000	0.5000	1.0000	0.2465

3. Cultural elements 一致性比例：0.0542；对"Evaluation index system of ecological structure of waterfront plant landscape in Xingqinggong Park, Xi'an"的权重：0.1571；λmax：4.1448

Cultural elements	Positive plant culture meanings	Plant growth years	Proportion of endangered and valuable plants	Plant cultural characteristics	Wi
Positive plant culture meanings	1.0000	6.0000	7.0000	3.0000	0.5646
Plant growth years	0.1667	1.0000	2.0000	0.2000	0.0836
Proportion of endangered and valuable plants	0.1429	0.5000	1.0000	0.1667	0.0547
Plant cultural characteristics	0.3333	5.0000	6.0000	1.0000	0.2971

4. Aesthetic elements 一致性比例：0.0366；对"Evaluation index system of ecological structure of waterfront plant landscape in Xingqinggong Park, Xi'an"的权重：0.2493；λmax：9.4271

Aesthetic elements	Plant shape and texture	Harmony between plants and water	Plant color diversity	Seasonal plant ornamental period	Plant neatness	Plant visual hierarchy	Plant aroma perception	Diversity of botanical art configurations	Plant peculiarities	Wi
Plant shape and texture	1.0000	0.3333	3.0000	0.5000	3.0000	2.0000	0.5000	0.5000	4.0000	0.1012
Harmony between plants and water	3.0000	1.0000	4.0000	3.0000	4.0000	3.0000	2.0000	3.0000	6.0000	0.2620
Plant color diversity	0.3333	0.2500	1.0000	0.3333	2.0000	0.5000	0.3333	0.3333	3.0000	0.0534

Seasonal plant ornamental period	2.0000	0.3333	3.0000	1.0000	3.0000	2.0000	0.5000	2.0000	4.0000	0.1381
Plant neatness	0.3333	0.2500	0.5000	0.3333	1.0000	0.5000	0.2500	0.3333	3.0000	0.0442
Plant visual hierarchy	0.5000	0.3333	2.0000	0.5000	2.0000	1.0000	0.3333	0.5000	4.0000	0.0744
Plant aroma perception	2.0000	0.5000	3.0000	2.0000	4.0000	3.0000	1.0000	2.0000	5.0000	0.1824
Diversity of botanical art configurations	2.0000	0.3333	3.0000	0.5000	3.0000	2.0000	0.5000	1.0000	4.0000	0.1182
Plant peculiarities	0.2500	0.1667	0.3333	0.2500	0.3333	0.2500	0.2000	0.2500	1.0000	0.0262
